# A modular, adaptable, and accessible implant kit for chronic electrophysiological recordings in rats

**DOI:** 10.1016/j.crmeth.2025.101146

**Published:** 2025-09-02

**Authors:** Raquel J. Ibáñez Alcalá, Andrea Y. Macias, Cory N. Heaton, Ricardo Sosa Jurado, Alexis A. Salcido, Neftali F. Reyes, Serina A. Batson, Luis D. Davila, Dirk W. Beck, Lara I. Rakocevic, Atanu Giri, Kenichiro Negishi, Sabrina M. Drammis, Ki A. Goosens, Travis M. Moschak, Alexander Friedman

**Affiliations:** 1 Department of Biological Sciences, University of Texas at El Paso, El Paso, TX, USA; 2 Computational Science Program, University of Texas at El Paso, El Paso, TX, USA; 3 National Institute on Drug Abuse, Baltimore, MD, USA; 4 Artificial Intelligence Laboratory, Department of Computer Science, Massachusetts Institute of Technology, Cambridge, MA, USA; 5 Departments of Psychiatry and Pharmacological Sciences, Center for Translational Medicine and Pharmacology, Friedman Brain Institute, Icahn School of Medicine at Mount Sinai, New York, NY, USA

**Keywords:** electrophysiology, modular, adjustable, kit, precision, 3D printed, chronic implant, electrode array, optogenetics, electrical, stimulation

## Abstract

Electrophysiological implants enable exploration of the relationship between neuronal activity and behavior. These technologies evolve rapidly, with multiple iterations of recording systems developed and utilized. Chronic implants must address a litany of complications, including retention of high signal-to-noise ratio in probes and the ability to withstand excess force over the experimental period. To overcome these issues, we designed a chronic implant for rats. Our comprehensive protocol optimizes the entire implant process, from assembling and testing the probes (Neuropixels) to implantation. In addition to addressing the complications previously mentioned, our implant can vertically adjust probes with micron precision and is constructed using modular components, allowing it to be easily modified for various research contexts, electrophysiological recording systems, headstages, and probe types.

## Highlights

• We develop a modular implant for electrophysiological recordings in rats
• The implant kit allows testing and calibration and facilitates implantation
• The kit enables vertical adjustment of probes with micron precision
• The implant is adaptable: e.g., optical fibers or different probe types may be added

## Motivation

The evolution of electrophysiological technologies and methods for recording extracellular electrophysiological data creates a need for adaptable and modifiable devices that can remain useful over time. Chronic implants that enable vertical movement of implantable probes enable researchers to search for strong neuronal activity within brain tissue over time. However, this must be done slowly to reduce irritation of the tissue. Our objective was to provide a comprehensive, open-source, and low-cost implantation kit to facilitate this process.

## Graphical abstract

**Figure:**
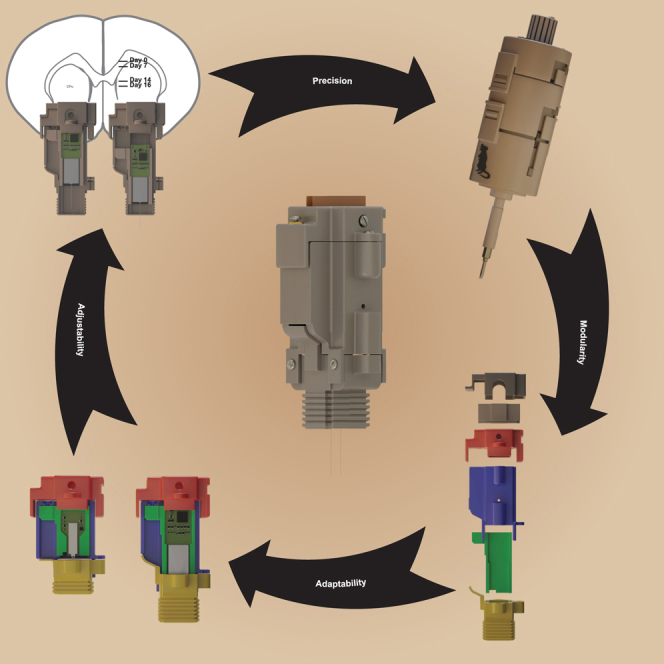

## Teaser

Ibáñez Alcalá et al. developed a highly adaptable implant, usable for chronic recordings in rats with high precision of brain targeting. Fundamental ideas that are implemented include modularity, precise vertical adjustment, adaptability to different recording systems, accessibility prioritization, and a comprehensive pipeline from building to implantation that could guide future implant development.

## Introduction

*In vivo* electrophysiological recording of neural activity in animal models is a critical tool for exploring the activity of different brain regions during various behaviors.^1^^,^^2^ The technology enabling these recordings has evolved over time.^3^^,^^4^ One early method for recording brain activity used a single microwire electrode in anesthetized animals.^3^^,^^5^ Further work enabled single-electrode neural recordings in awake, head-fixed animals.^6^ Recording technology then advanced with new probes (including electrode arrays,^7^^,^^8^ tetrodes,^9^^,^^10^ silicon probes, optical probes,^11^^,^^12^ and others^3^) and associated implants to record neuronal activity during animal behavior.^13^^,^^14^^,^^15^^,^^16^^,^^17^^,^^18^^,^^19^ Despite these advances, there remain several areas where they could be improved; including making precise implantation easier, reducing mechanical displacement of the implant or probes post-implantation, enabling implants to be compatible with different recording systems, and striking a balance between weight and durability. A major division across implants is whether they are designed for acute^1^^,^^20^^,^^21^ (used for a day or two) or chronic^21^^,^^22^ (left implanted for weeks or months) recordings. Chronic implants must account for additional factors like damage to the probe,^16^^,^^23^ inflammation from implantation,^24^^,^^25^ movement-related damage caused over time,^26^^,^^27^ scar tissue forming around the probes,^26^^,^^28^ and implant weight.^13^^,^^16^^,^^29^ These issues resulted in several variations in implant design and implantation protocols; for example, using more flexible materials when making probes,^30^^,^^31^ lowering probes slowly,^32^ or using ultrasonic vibrations^33^ while lowering the probe.

Implantation methods have been adapted; prioritizing/adding/altering features to be better suited for different uses.^34^^,^^35^ Some have been designed to prioritize light weight, thus having a minimal or nonexistent effect on the subjects’ behavior; these implants are generally developed for mice, with some providing modifications for their use with rats.^16^^,^^22^^,^^29^ Other implants are designed for maximum durability, particularly for rats; these implants are heavy.^17^^,^^36^ Other implants use a motorized mechanism for moving probes, adding weight to the implant and limiting usable probes.^15^^,^^19^ Certain implants add the capability of angling probes or placing them into an array for multiple recording sites.^17^^,^^18^ We wanted to create a durable implant for extended use in rats that is able to vertically adjust probes with micron-level precision, minimize lateral probe movement, be tractable to advances in probe/implant technology, accommodate different probe types, and be compatible with multiple recording systems while maintaining a high signal quality. We developed our implant to be easy to assemble and simple to adopt, using 3D-printed and commercially available parts. The implant is composed of modular components, facilitating easier modification and adaptation. We developed an accompanying implant kit with several accessories for implant construction, testing, and a custom screwdriver design that enables high-precision adjustment of probes post-implantation. We integrate inflammation-reducing inclinations including slow insertion^8^ and adjustment of the probe post-implantation. During the implantation surgery, we inserted the shank above the target depth, slowly reaching the target area over time.

## Results

### Implant kit overview

**Figure 1:**
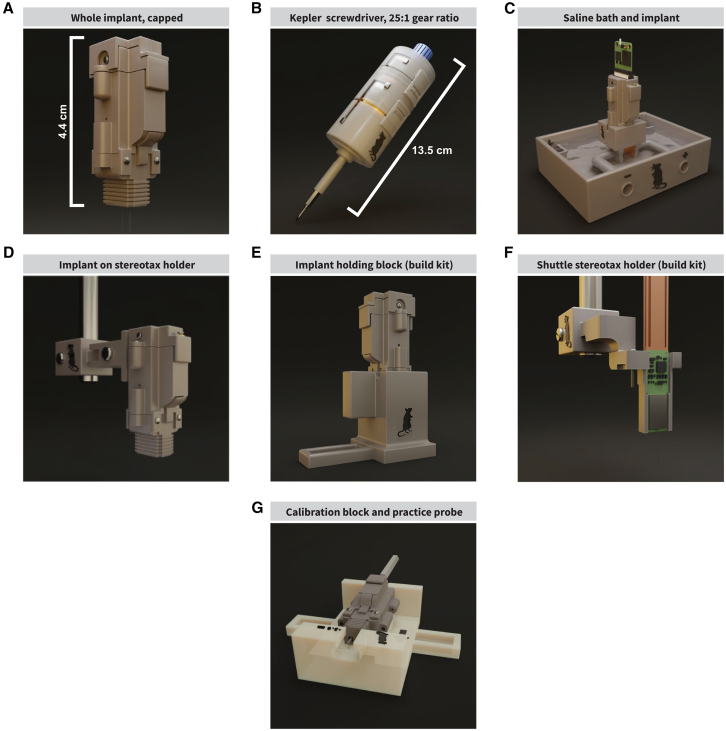
Kit overview (A) The implant is 3D-printed, easy to assemble, and features modularity to facilitate adaptation. It features a driving screw mechanism with a movable shuttle to adjust the vertical position of the probe(s) after implantation. (B) The Kepler screwdriver, a mechanical screwdriver with a gear system that enables micron-precision vertical adjustments to the probe’s position to keep track of location and protect the probe from damage as the probe moves through the tissue. Each turn at the input of the screwdriver results in a 25th of a turn at the output, allowing for a minimum of 0.012 mm adjustments with a 0.3 mm pitch screw. (C) A custom testing environment for evaluating gain and noise characteristics of the probe and acquisition system: a receptacle holds saline solution, stimulating electrodes, distal receptacles to hold ground wires, and a detachable implant block that holds the assembled implant over the saline. (D) The stereotaxic adapter holds the implant during surgery. (E) A large holding block as a part of the assembly kit to protect the probe(s) during assembly. If the probes are not retracted, the holding block protects the probe shanks in a channel. (F) The shuttle stereotactic adapter as part of the assembly kit is used to lower the fragile probes into the implant. The shuttle is held in place with the drive screw used to adjust the probe’s vertical position. (G) Because 3D printing may introduce some small errors in the vertical dimensions of the implant, implants are calibrated after assembly. We created an easy-to-use calibration block and protocol, along with a dummy probe and shuttle for practicing assembly and calibration.

We created an implant kit designed for chronic *in vivo* electrophysiological recordings in rats. The implant can be constructed and assembled completely “in house” with higher-end consumer-grade 3D printers and commonly available parts. The implant is simple to construct and can be built in 2 days, including 3D-printing, washing, curing, and adhesive curing of individual components. The implant with 2 probes weighs ∼8.4 g (Figure 1A). When paired with our custom-designed screwdriver, probes can be vertically adjusted with micron precision (Figure 1B). Further, probes can be tested early in the assembly process (Figure 1C). The implant was designed to be small, lightweight, and durable enough to withstand chronic use in rats (Figure 1D). The implant can be built using a stereotax holder plus holding block, protecting the probes (Figures 1E and 1F). Our implant is compatible with different headstage platforms and easily modified for different probes. There is also a setup provided for calibrating implants to maintain consistency and account for variance introduced by 3D-printing (Figure 1G). The shuttle shown is designed for use with Neuropixels 1.0 silicon shanks,^37^ with an alternative design for Neuropixels 2.0^38^ provided as well. Overall, simplicity, adaptability, durability, accessibility, and precision are all important factors implemented in our implant design.

### Implant components and reasoning behind design choices

**Figure 2:**
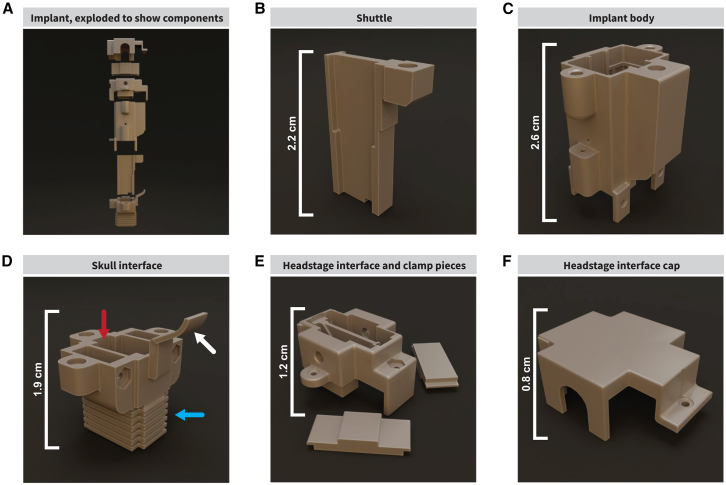
Implant components (A) Our modular implant consists of 5 main parts. (B) The shuttle holds one/two probes and moves on the vertical axis (*z*) with a screw mechanism via a threaded insert. (C) The implant body holds the shuttle in place on an internal rail to prevent it from moving in any unintended axis. The drive screw is inserted and turned from the top. It is reinforced with an internal grid pattern to protect probes from damage originating from rat behaviors. Ground wires are threaded from the bottom and inserted into the implant body. (D) The skull interface features a rail (red arrow) to guide the shuttle. It has two holes on the bottom for the probe shanks. When assembled, the cover piece on the side (white arrow) seals the screw channel to protect the drive screw shaft and shuttle from contaminants. The bottom ridges (blue arrow) allow cement to adhere to the implant. (E) The headstage-agnostic interface allows the implant to be used with most acquisition systems. The probes’ ZIF connectors come out of the top of the piece and are held in place by a “biting” mechanism consisting of two removable “teeth.” (F) The headstage interface cap covers the top of the implant to protect the ZIFs from contaminants.

The implant consists of five modular components, allowing the implant to be tailored to different research requirements (Figures 2A and S1A). The shuttle component holds the probes within the implant and can be altered to change the number/types of probes (Figures 2B and S1B). The shuttle moves the probes vertically via a driving mechanism using a threaded insert. This vertical movement is possible by using a fine screw (space between threads [*pitch*] ≤ 0.5 mm; we used a 0.3 mm thread pitch to prevent “thread skipping”) in the driving mechanism, which allows for fine movement along the vertical axis, important for reducing tissue damage and maintaining a high signal-to-noise ratio. The shuttle is held in place by a “rail” inside of the implant body (Figure 2C) and is protected with an internal molded grid pattern (Figure S1C). The rail continues into the skull interface (Figures 2D and S1D) to stabilize the shuttle. This prevents lateral movement, improving stability and reducing sheer forces on the probe shank, preventing breakage. The friction of the rail, along with the drive mechanism, prevents unwanted movement along the vertical axis, reducing drift. We designed a headstage interface that is compatible with multiple recording systems including the Neuropixels Data Acquisition system^39^ and the Open Ephys ONIX system^40^; made possible by leaving the probes’ zero insertion force (ZIF) connectors exposed at the top of the headstage interface so that any device can be connected through a compatible circuit connector (Figure 2E). The implant can be capped to protect the ribbons (Figure 2F). To protect the probe from pulling movement at the ZIF connectors and prevent the probes from unintentional movement, the extra length of the probe ribbon is folded into an “S” shape and tucked inside the implant. The ZIF connectors are threaded through two 0.5 mm slots at the bottom of the headstage interface, covering the inside of the implant. The headstage interface piece has a “biting” mechanism holding ZIF connectors in place with two screws mounted onto the piece (Figures S1E and S1F). We recorded two rats for months at a time (one for 112 days and the other for 64 days). To use a different system, the interface piece can be altered to have an enclosure suitable for that recording system.

### Custom screwdriver enables vertical movement of probes with micron precision

**Figure 3:**
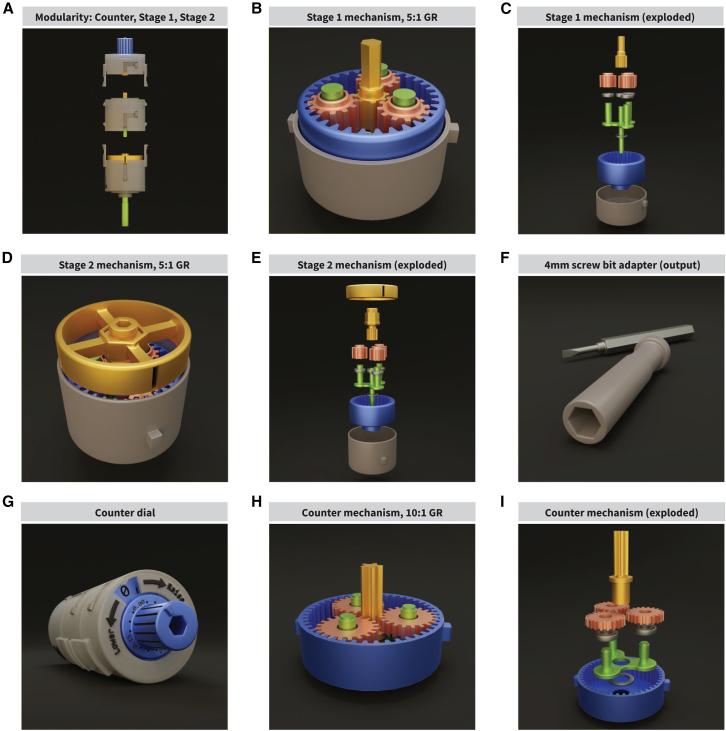
The Kepler screwdriver (A) The “Kepler screwdriver”/“Kepler driver” is a modular, 2-stage mechanical gear screwdriver that converts one rotation at the input into one 25th of a turn at the output. For a drive screw of pitch = 0.3 mm, one turn of the Kepler driver would result in the shuttle component of the implant to move 0.012 mm along the shaft of the screw. (B–E) The Kepler driver’s first (B and C) and second (D and E) gear stages are a planetary gear system with a GR of 5:1. Because the first stage feeds into the second stage, the two stages’ GRs are multiplied together, resulting in a GR of 25:1. Their input is located at the central sun gear (yellow), and the output is on the carrier (green), which rotates within the stationary ring gear (blue), driven by three planetary gears (pink). Each gear mechanism is held in a case (gray) that holds the ring gear in place. The main differences between the two stages are the sun gear, which has a hexagonal shaft on stage 1, and a hex socket at stage 2 (C and E). Stage 2 has an added ring indicator that simply follows the movement of the sun gear to show movement. (F) The second-stage carrier connects to the screwdriver bit adapter, which was originally made with a 4 mm hex socket for small bits; it can be easily re-sized or replaced if needed. (G) To prevent errors when counting turns, we designed a detachable counter module. The gear system does not alter the turn rate of the input; rather, it passes the turns directly down to the first gear stage of the screwdriver. The counter’s gear system output turns a disk that indicates the number of turns completed, with the counter dial indicating fractions of a turn. (H and I) Similar to the other gear systems on the screwdriver, the counter features a planetary gear system with a GR of 10:1; a whole rotation of the input is converted into a 10th of a rotation at the output. The input to the system is the sun gear, whereas the output is the carrier. Unlike the other gear systems, the sun gear passes through the carrier to drive the input rotations directly onto the next gear system so that the rest of the mechanism’s turn rate is not affected.

The number of rotations by the driving screw in the implant’s movement mechanism controls the distance and speed the shuttle travels. When the distance traveled per 360-degree rotation is equal to the pitch of the drive screw, sub-dividing the rotations divides the distance proportionally (see STAR Methods “calculating vertical probe location”). If the driving screw’s pitch is 0.3 mm, then a full turn makes the shuttle travel 0.3 mm, half a turn 0.15 mm, and a quarter turn 0.075 mm. Such granularity slows the shuttle, preventing excessive stress on the probe shank as it moves through tissue. This allows the estimation of the shank’s position. It is difficult to know exactly how far and fast a small screw is rotated by hand. For this reason, we designed the *Kepler screwdriver* (Figure 3A), a precision mechanical screwdriver with concentric cascading planetary gears (Figures 3B–3E) with a gear ratio (GR) of 5:1, for a total of 25:1, meaning 25 full turns at the input of the system completes 1 rotation at the output (see STAR Methods “the Kepler screwdriver”). The distance traveled by the shuttle would be 0.012 mm per full turn of the Kepler driver. The Kepler screwdriver can be modified to change screwdriver bit compatibility (Figure 3F) and limits how fast the screw can be turned to help protect the probes. An optional mechanical counter attachment limits human error when counting turns (Figure 3G). The counter attachment tracks input rotations with a 10:1 planetary gear. This planetary gear rotates a disk with numerical etchings while fractions of a rotation are displayed at the knob (Figures 3H and 3I). The GR of the attachment is not counted toward the mechanism’s total GR and attaches at the top of the device, highlighting the modular design of the implant. Instructions on how to 3D-print and assemble the Kepler screwdriver are in GitHub (Zenodo: https://doi.org/10.5281/zenodo.16126335).

### Comprehensive build kit simplifies implant assembly, protects probes, and aids in implantation surgery

**Figure 4:**
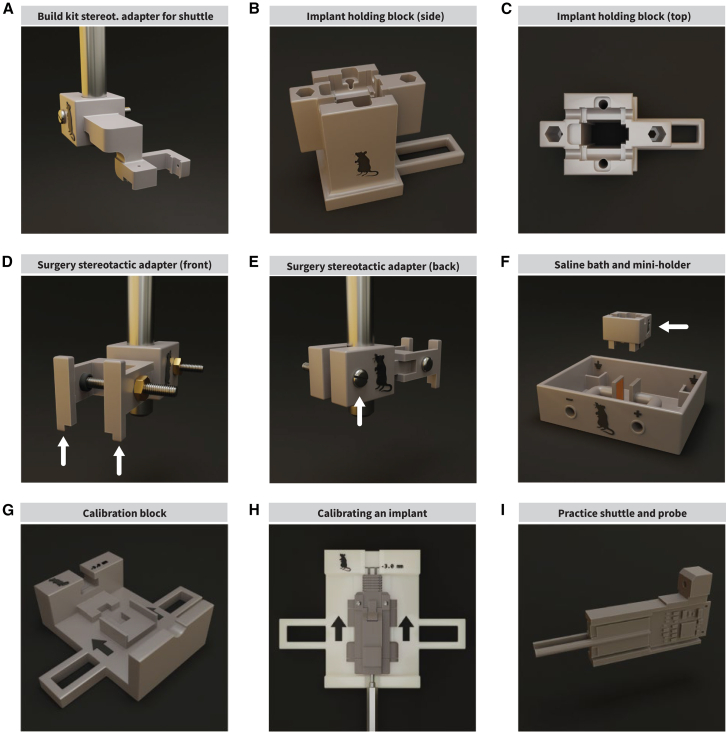
Assembly, surgery, calibration, and testing accessories (A) The shuttle stereotactic adapter is designed to hold one implant shuttle piece loaded with a probe(s). It leverages the three-coordinate system of a stereotaxic device to maneuver the shuttle and probes into position and mount them onto the implant. The adapter block attaches to a standard stereotaxic holder and has a screw and nut to tighten the adapter around the holder. (B and C) The implant holding block is designed to hold the skull connector piece for implant assembly. The holding block has a tall, empty channel down the center to protect the probes’ shanks from damage when not fully retracted. The two hex sockets can hold a 4 mm screwdriver bit each. The tail serves as an additional anchor point. (D and E) To hold a fully assembled implant during surgery, we created the implant stereotactic adapter. The adapter “arms” (D, arrows) slide onto the implant and hold it by the headstage interface piece. The screw and nut ensure that the implant is held firmly in place. Similarly, the screw and nut on the adapter (E, arrow) ensure that the adapter does not slide off the stereotactic device. (F) For testing probes pre-implantation, we designed a saline bath consisting of a receptacle to hold saline, two smaller receptacles for the probes’ ground wires/electrodes, and two electrode plates that flank the probe shank while it is in the saline to apply a voltage across them for noise and gain testing. The mini holding block (arrow) holds a fully assembled implant and is detachable. (G) Implant calibration ensures that repositioning the probes is repeatable and precise by considering 3D printing errors that affect the implant’s inner guide channel. We developed a calibration block to visualize and correct errors. The goal of calibration is to identify a starting position for the probe so that repositioning happens from a known location and successive adjustments result in expected depth. (H) Implants are calibrated when 10 drive screw turns result in a 3 mm shank tip displacement out of the base of the implant and the shank tip aligns with the reference line on the calibration block. The probe shank pictured belongs to the practice probe in (I). (I) We provide a test shuttle with simulated probe shanks to practice calibration or implant assembly.

Electrode arrays are typically expensive and fragile. If a shank is damaged, the entire probe becomes unusable. This is a challenge when loading the probes into the implant and throughout assembly. To minimize the risk of the probes breaking, we developed an assembly kit and protocol to make the process accessible (Zenodo: https://doi.org/10.5281/zenodo.16126335). The washing, post-processing, and curing of the 3D-printed pieces; assembly; and adhesive drying can be completed in 4 h. We developed an adapter so that a stereotax can be used when loading probes (Figure 4A); this allows probes to be maneuvered safely and lowered into place with the optional aid of a surgical microscope/magnifying glass to monitor shanks (Figures S2A–S2C). The shuttle and probes are then lowered into the skull interface and held in place by our custom-designed holding block (Figures 4B and 4C), protecting the shanks. The implant assembly can be completed with the two implant pieces on the block. The probes can be retracted into the implant, shielding them from accidents. The implant can remain on the holding block until it is implanted. For surgery, we developed a stereotaxic adapter to hold the completed implant (Figures 4D, 4E, and S2D,) which keeps the implant secure during implantation.

Our implant kit includes a tool for testing probes before implantation. This can give insight into the electrical characteristics of the probes and system they are connected to while ensuring the probes work after assembly. For Neuropixels probes, it is recommended to test for gain and noise by dipping the shank into saline and initiating a recording as a troubleshooting step alongside software self-tests. We designed a 3D-printed saline bath that features a tray that holds saline, two copper plates flanking the electrode shank, and small receptacles distal to the probes for the ground (Figure 4F). To hold the implant above the saline, a separate miniaturized holding block was built, allowing the adaptation of the bath to different implant types or to test the probes themselves on a stereotaxic device (Figures S2E and S2F). This saline bath allows for noise and gain testing on the probes before implantation (see STAR Methods “probe testing and calibration”). Before implantation, the implant is calibrated to ensure reproducibility of probe repositioning. First, the assembled implant is placed on a custom-made calibration block (Figures 4G and 4H). The drive screw is turned 10 times to displace the probe 3 mm. Second, the probe is displaced again until the tip aligns with the 3 mm reference mark on the calibration block while counting the turns; this corrects for the error introduced by tolerances and printing errors. The probe is retracted, and the drive screw is turned the number of times counted in the second step. Finally, the screw is turned 10 times again to test if the tip aligns with the mark. If not, the process is repeated (Figures S2G–S2I). Both assembly and calibration can be performed with a practice shuttle and mock probes (Figure 4I).

### Implantation surgery

**Figure 5:**
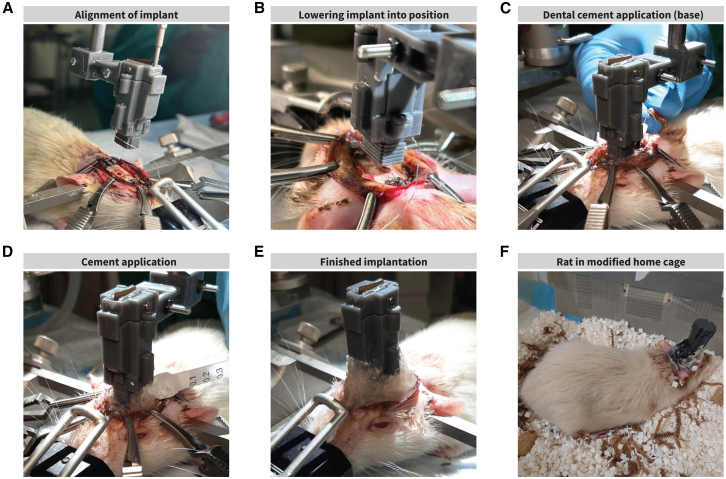
Surgical procedures (A) Implant alignment on the mediolateral (ML) and anterior-posterior (AP) axes to implant the probes near the dorsomedial striatum. The probe is implanted at a known depth, accounting for initial target depth, skull thickness, and implant wall thickness. The amount of drive screw turns to reach this length is calculated using Equation 1 (see “distance as a function of drive screw turns”). (B) With the probe drawn at the appropriate length, the implant is lowered onto its final position on the dorsal-ventral axis (DV). This is done slowly, watching for buckling with the help of a surgical microscope. The tip of the shank is “bounced” on the brain tissue a few times to test for buckling; if buckling is observed, the tissue is re-hydrated with saline and dexamethasone. (C) After the implant has been lowered onto the skull and the probe inserted, sterile Vaseline is applied to the bottom of the implant to prevent blood from entering the implant. Metabond is applied to the bone, screws, and base of the implant to create a foundation for the implant and strengthen adhesion of dental cement, as well as insulating the ground screws from the surrounding tissue. (D) Dental cement is added to the Metabond on the base of the implant up until the skull connector. (E) Excess tissue around the implantation area is cauterized using silver nitrate. Probe is post-surgically adjusted using the Kepler driver (0.12 mm, or 10 turns with a screw pitch of 0.3 mm). The number of turns is calculated using Equation 2 (see “distance as a function of drive screw turns”). (F) The rat is removed from the stereotactic device, and post-surgical care is administered. A topical antibacterial antifungal corticosteroid cream is applied, and Enrofloxacin (antibiotic) with Meloxicam (NSAID) in 10mL Ringer’s solution is injected to reduce the chance of complications during recovery (see Methods S1). The rat is placed in a modified cage. Figure depicts a rat weighing ∼500 g.

For surgery, the implant is mounted onto a holding piece that can be connected to a stereotax (Figures 5A and 5B, see STAR Methods “surgical and post-surgical procedures”). To maximize the lifespan of implanted probes, several measures were taken. The implant was affixed to the rat’s head in the most stable configuration possible to prevent damage from rat behavior. To reduce this damage, we cleaned the skull surface of connective tissue, scored the skull, and added eight anchor screws (Figures S3A–S3G) around the implant, which were covered in dental cement to make a strong foundation. These screws and dental cement foundation keep the implant in place during behavioral manipulations (Figures 5C and 5D). After the probes are set within the tissue, applying Vaseline around the base of the implant (Figure S3H) can prevent discharge and blood (which could obstruct movement along the shuttle rails) from entering the body of the implant. At the end of the surgery, we sutured the skin at the anterior and posterior skin-implant interfaces and cauterized excess tissue around the implant with silver nitrate to prevent tissue from regenerating (Figures 5E, S3I, and S3J). Post-surgical care is important to prevent implant failure. To prevent infections, animals were given routine broad-spectrum antibiotic injections (enrofloxacin). To reduce itching and to locally disinfect the surgical site, we routinely applied hyaluronic acid and chlorhexidine on live tissue around the implantation site (Figure S3K). A combination of nystatin, neomycin, thiostrepton, and triamcinolone can also be used to treat the area. Modifications to the home cage can prevent damage to the implant and increase comfort; in this case, we provided a larger/taller home cage space with minimal protrusions from the walls, reducing the areas the implant could catch on (Figure 5F). We provide all information related to the modified home cages in Methods S1. Despite these modifications, the rat could run violently into the walls of the cage, vibrating the probes enough to move or break them. Shifts in probe location may be identifiable through the presence of drift artifacts, which we have not yet observed within our implanted animals. The implant itself is designed to minimize probe movement with tight tolerances between the internal rail and shuttle; the drive screw is held in place by the headstage interface piece, which exerts downward force to prevent the drive screw from traveling upwards.

### Probe alignment, lowering to initial location, and vertical probe adjustment post-surgery

One method for preventing probes from breaking is lowering them slowly while monitoring the shanks for buckling. We lower the implant slowly, raising it back up if needed, until we are sure that the shanks are inserted with minimal force. If the shanks buckle on the tissue, it may be due to drying/hardening tissue, rectified by hydrating the tissue with a drop or two of 4 μg/mL dexamethasone. The probe shanks are only drawn after the surgical site is ready for the implant to be lowered onto the skull. The probes are at risk of breaking immediately after surgery due to tissue inflammation, and at later points because of scar tissue formation around the shank; inflammation and scar tissue can also reduce recording quality over time. To minimize the dangers of moving a fragile object through scar tissue, we leveraged the adjustable nature of the movable shuttle by making micro-adjustments using our Kepler screwdriver post-surgery (Figure S3L). With this approach, we slightly adjusted the position of the probe shank within the tissue in a slow and controlled manner to avoid breaking the probe shank and tracking the depth of the shank tip after each adjustment. To keep track of where the tips of the probe shanks are, we first must implant the probes at a known depth. To do this, we first measure the thickness of the skull with calipers, then draw the probes out of the implant to that distance plus the thickness of the skull interface’s bottom wall (0.5 mm). We next carefully lower the implant into place with the help of a stereotax while monitoring the probe shanks for buckling using a surgical microscope. Once the probes are implanted, they are further inserted into the brain (100–300 μm) post-surgery, and with our precise micro-adjustment protocol, we can track what depth the probes are located at after each adjustment. Further details about this process can be found in the STAR Methods (“calculating vertical probe location” and “probe location during and after surgery”). Using this method, we reached the dorsal striatum (−3.45 mm DV) within 3 weeks when making 100–300 μm adjustments 2–3 times per week. After the target was reached, 60 μm adjustments were made every other week (see STAR Methods “post-surgical micro-adjustments”). With the Kepler screwdriver, moving the probe 3 mm takes 25 turns (12 μm per turn). We lowered probes slowly during post-surgical adjustments (5 s per turn, or 12 turns per minute) to reduce inflammation (Figure S6) and reduce the pressure put on the probe. Rats were anesthetized for probe movement sessions. If faster vertical probe adjustments are needed, the gear ratio of the Kepler screwdriver can be lowered, allowing the shuttle to travel farther. In contrast, slower probe movement with a higher gear ratio could be used, though it may be more feasible using a stepper motor and programmable electronics instead of manually turning a screwdriver.

### Implant grounding considerations

In electronic systems, ground is the reference point from where voltage is measured. If the ground circuit is conducting a noise signal, that noise will be added onto the recorded signal and amplified by the recording equipment; this can overpower lower amplitude (roughly 10–100 μV) extracellular recordings or saturate the signal-processing hardware, impeding signal analysis techniques. In our surgical protocol, we address this by grounding our probes as far away as possible from electrically active tissue and avoiding interconnecting probes to prevent ground loops. Because muscle tissue is electrically active (producing signals in the 1–100 mV range), it is important that the implantation area is cleared of muscle connective tissue during surgery. Proper grounding is critically important for avoiding ground loops since they may introduce 50–60 Hz electromagnetic interference artifacts (see STAR Methods “grounding considerations”).

### Electrophysiology recordings from the implant

**Figure 6:**
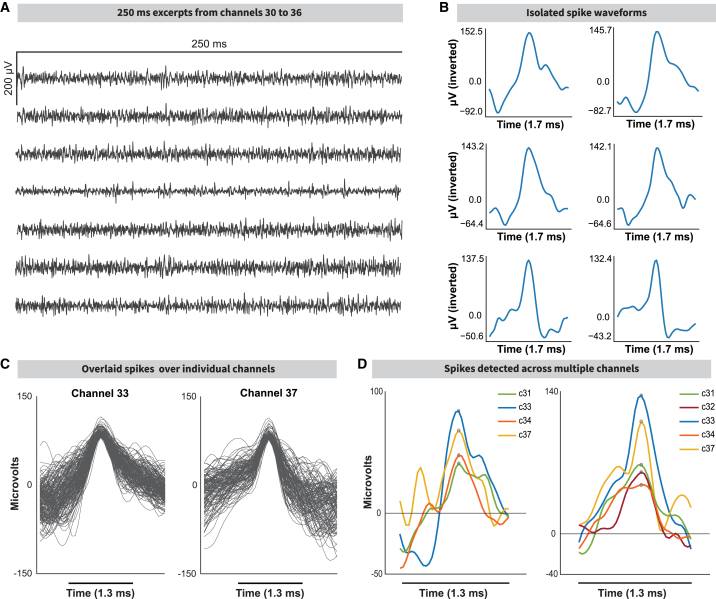
Stable recordings 75 days after implantation (A) Continuous recording of extracellular activity from a single Neuropixels 1.0 probe implanted at coordinates −1.2 mm AP, +2.5 mm ML (from Bregma). The recording was made the day after the probe was driven −60 μm DV during a post-surgical probe adjustment 75 days after implantation, leaving the tip of the probe situated at −2.74 mm DV. (B) Isolated spike examples from (A). (C) Average spike waveform examples. (D) Spike waveforms present across neighboring channels. The general spike shape is conserved across a few physically proximal electrodes, but the spike amplitude is reduced further from the “main” electrode where the spike is recorded.

With our implant, we recorded two rats using the Neuropixels Data Acquisition system and the Open Ephys GUI. One was recorded over 64 days, the other over 112 days (see STAR Methods “recording and signal processing”). We achieved clear recordings with a high signal-to-noise ratio (Figure 6A, channels 30–36). There was minimal detectable lateral movement of the shuttle after assembly prior to surgery or drift during electrophysiological recordings. We were able to extract individual spike waveforms in MATLAB (Figures 6B, 6C, S4A, and S4B) and spatially relate them across a varying number of channels (Figures 6D and S4C). We obtained recordings on days 75, 76, 81, and 82 after surgery, repositioning the probe by 60 μm before recording on days 75 and 81. Signals were pre-processed, and recording quality was relatively consistent (Figure S4D; see STAR Methods “signal pre-processing” and “spike detection”).

### Proof-of-concept modifications to the implant

**Figure 7:**
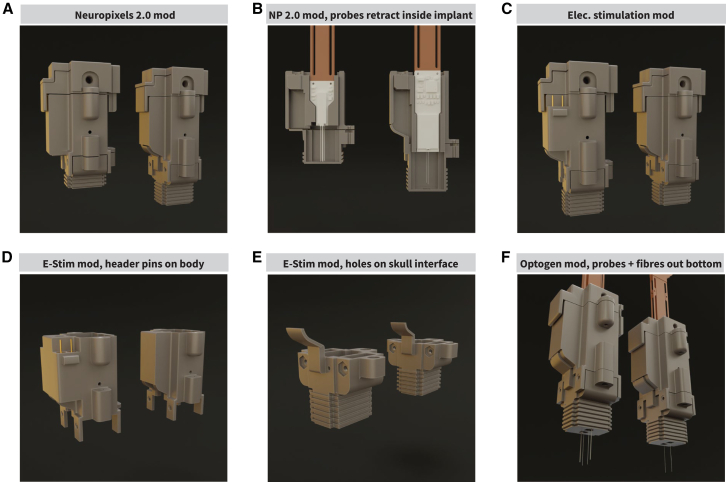
Modularity facilitates modifications (A) Side-by-side comparison between an adaptation of the implant for Neuropixels 2.0 (NP2.0) probes (left) and the original (right). Only the shuttle (not pictured), implant body, and skull interface have been modified, leaving the headstage interface and cap untouched. The modifications led to an 8 mm reduction in height and were completed over the 2 days. (B) A bisected view of both the NP2.0 (left) and original (right) implants. (C) Side-by-side comparison between the electrical stimulation modification (left) and the original implant (right). This modification adds the ability to mount stimulation electrodes alongside the probes. (D and E) Only the implant body (D) and skull interface (E) were modified. A two-pin header receptacle was added to the implant body on either side of the drive screw rail. A wire would be soldered to each header pin, one for the cathode (−) and one for the anode (+) of the stimulation electrodes. These wires travel down into the skull interface piece. Two holes on the side of the skull interface allow the wire to be fed into the skull interface, and the wires would be soldered onto stimulation electrodes. (F) The final proof-of-concept modification to the implant for optogenetics. Static optical fibers are mounted alongside the probes, offset 1.25 mm to the side, and 0.5 mm above the tip of the probe. A fiber cannula receptacle was created on either side of the skull interface. These receptacles hold one cannula each, through which an optical fiber is threaded. Similarly, the headstage interface also has cannulas to allow optogenetic stimulation devices to be connected.

To demonstrate the modifiable nature of our implant, we have designed proof-of-concepts of alternative use cases (see STAR Methods “implant modifiability”). We designed a prototype for using Neuropixels 2.0 probes instead of the Neuropixels 1.0 probes (Figures 7A, 7B, and S5A–S5D), achieved by changing the shuttle piece to accommodate different probe shapes (Figure S5A). We demonstrate how adding pin headers and connectors to the implant body and electrodes to the skull interface enables electrical stimulation functionality (Figures 7C–7E and S5E). Changing the skull and headstage interface pieces to fit optical fiber cannulas allows for the addition of optical fibers, enabling closed-loop optogenetic manipulation along with electrophysiological recording (Figures 7F, S5F, and S5G). We include two optical fibers situated 500 μm above the electrode probe tip.

### Immunostaining for signs of inflammation

To examine a form of immune response in the brain tissue after implantation, we implanted a Neuropixels 1.0 probe into a rat and followed our gradual adjustment protocol (the probe was repositioned from initial insertion to target site [DV = −5.0 mm] over 16 days, see STAR Methods “inflammation assessment”). The implant was left for an additional 7 days after the probe reached the target site (23 days total), then immunohistochemical analysis was performed *ex vivo*. After imaging, we see dense expression of the CD11b+c marker around the surgery and initial probe insertion site; in comparison, expression of the CD11b+c marker is less prevalent closer to the target site (Figure S6). This suggests that the slow and gradual movement of the probe during surgery and repositioning to the target site over time can help ameliorate the immune response, which aligns with other research on implantation in brain tissue.^32^^,^^41^

## Discussion

One example of an additional feature could be implemented by altering the implant-skull interface component to customize it for each subject. In monkeys, this was done by scanning the skull before printing the interface,^42^^,^^43^ and alternatively, flexible materials that adjust to different skull shapes could be used. A better seal would increase headstage stability with less cement, reducing implant weight. Another idea used in non-human primate models is moving the shuttle to different recording locations within the implant,^44^^,^^45^ allowing access to a wider region of interest. A miniaturized, less durable version of the implant could be created. We designed this implant to be durable at the cost of weight to use with rats, which are more likely to damage the implant with excessive force, that were self-administering oxycodone. These future directions and developments aim to improve the utility of the implant and ultimately allow this implant platform to be suitable for various research contexts.

The principles underlying the implant kit, including easy assembly, modularity, highly precise vertical adjustment of probes, adaptability to different recording systems, prioritization of accessibility, and the provision of a comprehensive pipeline for building, testing, and implantation are design considerations that could be useful when developing future implants as electrophysiological recording technologies advance. The implant was also designed to have wide appeal for researchers who use rat models to explore neurological activity during behavioral performance.

### Limitations of the study

The implant is larger and heavier than some other chronic implant options,^22^^,^^46^ and while the weight could be lowered with different materials or using smaller probes (e.g., using Neuropixels 2.0 probes, example provided in Figure S6), in subjects that receive certain substances of abuse, durability would remain a key factor to consider. The size of the bottom of the skull interface (measuring 8.3 × 11.6 mm) limits the utility of the implant to research using adult rats, though this can also be addressed by using smaller probes and modification of its dimensions. Although our implant was not intended for or tested on mice, we recognize that its weight (about 8.4 gr with one Neuropixels 1.0 probe) is a limiting factor to its use in mouse studies. However, an implant made for mice requires less strict durability requirements than rat implants do; thus, the implant’s wall thickness (0.5 mm) or build material could be revised.

Perturbations in the brain tissue related to the chronic presence of an electrode array within the brain tissue trigger the accumulation of microglia and astrocytes at the electrode/brain interface.^4^^,^^41^^,^^47^ This reaction disturbs the distribution of neurons and non-neuronal cell types and can result in gradual neurodegeneration at the implantation site leading to reduced extracellular signal quality.^48^ Although studies that relate all of impedance, immune response, and signal quality are rare, there is some evidence that tentatively suggests that the presence of a “glial scar” relates to increased impedance at the electrode/brain interface and may reduce the quality of recorded signals,^47^ especially when attempting to isolate single-unit activity. However, the relationship between impedance and signal quality is complex, with many factors affecting impedance measurements.^47^^,^^49^ Nevertheless, it is reasonable to hypothesize that repositioning the probe could improve signal quality, if not by moving to an unscarred position, by moving closer to a neuron/neuron population. Movable probe implants allow for recordings of novel regions across successive sessions; this can be desirable when tracking or searching for single neuronal activity over time.^36^ These ideas, either in isolation or in conjunction, could have triggered an increase in implant designs that feature microdrives.^17^^,^^35^^,^^36^^,^^41^

During probe insertion into the tissue, the final stages must be done without observing the probe. This could result in probe buckling not apparent until recording or even until the probe is extracted, which could cause the implant to reach a non-targeted region. To minimize this risk, we removed the dura mater during implantation, making penetrating the softer tissue easier. We changed the angle of the microscope to observe the probe as long as possible. Slow insertion (around 10 s per mm) was implemented to ensure the probe enters smoothly and allow for readjustment. Post-surgical software tests, (within Spike GLX and the Open Ephys GUI) like the shift register test, were run to check the probe’s integrity and position. Additionally, the two probes we recovered (despite the implant not explicitly designed with probe recovery) demonstrated no signs of buckling or other loss of integrity. Going forward, tools like online recording could be implemented into the workflow to gauge the probe’s integrity and help increase implantation precision.

While constructing the implant, we did not account for probe removal and recovery following implant use because implants remain in use for months at a time and multiple connection cycles increase the chance of damaging the Neuropixel’s probes’ ZIF connectors. Despite this, it is possible to recover the probes by gently cracking open the implant using a Dremel. Redesigning the implant by moving these screws could allow for the implant to be opened with a screwdriver for easier probe recovery. Moreover, the design choice to not house the headstage(s) within the implant does put the fragile headstage electronics and ZIF connectors at risk; however, a headstage-agnostic mating method increases the implant’s cross-compatibility with different recording systems, for example the Neuropixels Data Acquistion system and the Open Ephys ONIX system, which both feature headstages with different form factors. This targets a specific niche that aims to increase the implant’s usefulness regardless of the recording equipment a neuroscience lab has access to. In the case where headstage protection is indispensable, the modularity of our implant enables the experimenter to design and replace the headstage interface piece with a housing piece.

Modification to the shuttle piece, rail, and skull interface would also be necessary to target more lateral structures of the brain (larger than the 1.5 mm medio-lateral the implant is originally designed for), which may limit its use further. For targeting more medial structures, we believe that only the shuttle piece would need to be modified to reduce probe separation down to 2 mm (1 mm from the zero medial coordinate) by reducing the middle wall of the shuttle to 0.5 mm. The depth of implantation is limited by the length of the probe shank (10 mm) minus 0.5 mm to account for the thickness of the bottom wall of the skull connector; however, this is usually sufficient for both rat and mouse brains. Finally, the current implant design can realistically only fit two probes (one on each side); adding more probes may require modifications to the overall implant size, which would be dependent on the size and weight of the probes used.

## Resource availability

### Lead contact

Further information and requests for resources and reagents should be directed to and will be fulfilled by the lead contact, Alexander Friedman (afriedman@utep.edu).

### Materials availability

All 3D files reported in this paper have been deposited in GitHub (Zenodo: https://doi.org/10.5281/zenodo.16126335).

### Data and code availability

• All data reported in this paper will be shared by the lead contact upon request.
• The code used to analyze the data reported in Figures 6 and S4 has been deposited in GitHub (Zenodo: https://doi.org/10.5281/zenodo.16288405, under “Porting Open Ephys”). The code and pre-processed data used to visualize Figures 6B, S4A, and S4D have been deposited in GitHub (Zenodo: https://doi.org/10.5281/zenodo.16126335).
• Any additional information required to reanalyze the data reported in this work paper is available from the lead contact upon request.

## Acknowledgments

This project was supported by the NSF/CAREER (#2235858), NIH/NIDA (#R01DA058653), U-RISE T34GM145529, G-RISE T32GM144919, and 1R25GM132959-05. The authors also thank Mia Maestas and Eduardo Montanez for their valuable contributions to histological analysis.

## Author contributions

Conceptualization, R.J.I.A. and A.F.; data curation, R.J.I.A., L.D.D., and A.F.; formal analysis, R.J.I.A., R.S.J., L.D.D., D.W.B., L.I.R., A.G., S.M.D., and K.N.; funding acquisition, K.A.G., T.M.M., and A.F.; investigation, R.J.I.A., A.Y.M., R.S.J., A.A.S., N.F.R., D.W.B., L.I.R., S.M.D., and K.N.; methodology, R.J.I.A., A.Y.M., R.S.J., A.A.S., N.F.R., T.M.M., and A.F.; project administration, T.M.M. and A.F.; software, R.J.I.A., L.D.D., L.I.R., D.W.B., and A.G.; supervision, T.M.M. and A.F.; validation, R.J.I.A., A.Y.M., R.S.J., and L.D.D.; visualization, R.J.I.A., A.A.S., N.F.R., L.D.D., and A.F.; writing – original draft, R.J.I.A., C.N.H., A.Y.M., R.S.J., and A.F.; writing – review & editing, R.J.I.A., C.N.H., K.A.G., T.M.M, and A.F.

## Declaration of interests

The authors declare no competing interests.

## STAR★Methods

### Key resources table

**Table:** 

REAGENT or RESOURCE	SOURCE	IDENTIFIER
**Antibodies**		

Anti-CD11b+c	Abcam	Cat# ab1211; RRID:AB_442947
A546 conjugated donkey anti-mouse IgG	Thermo Fisher Scientific	Cat# A32787TR; RRID:AB_2866494
NeuroTrace 435/455	Thermo Fisher Scientific	Cat# N21479

**Deposited data**		

STL files and Blend files	This paper	Zenodo: https://doi.org/10.5281/zenodo.16126336

**Experimental models: Organisms/strains**		

Sprague-Dawley Rats (CD® IGS) Crl:CD(SD)	Charles River Laboratory	RRID:RGD_734476

**Software and algorithms**		

Signal processing scripts	This paper	Zenodo: https://doi.org/10.5281/zenodo.16288405
Blender 3.4.0	Blender	https://download.blender.org/release/Blender3.4/
Preform	Formlabs	https://formlabs.com/software/preform/
Spike GLX	Github	Github: https://billkarsh.github.io/SpikeGLX/
Open Ephys GUI	Open Ephys	https://open-ephys.org/gui
Blender 3.4.0	Blender	https://download.blender.org/release/Blender3.4/
Preform	Formlabs	https://formlabs.com/software/preform/
Spike GLX	Github	https://billkarsh.github.io/SpikeGLX/
Open Ephys GUI	Open Ephys	https://open-ephys.org/gui

**Other**		

M1x4 screw	McMaster Carr	91430A151
M1.4x12 screw (0.3 mm pitch)	McMaster Carr	91800A711
M1.4 x 4 screw	McMaster Carr	91430A156
M1 threaded insert	McMaster Carr	92120A110
M1.4 threaded insert	McMaster Carr	92120A140
M1 nut	McMaster Carr	90591A311
M2.6 x 25 screw	McMaster Carr	90353A120
M2.6 nut	McMaster Carr	90592A008
Drill bit 1.0 mm	McMaster Carr	2958A25
Drill bit 1.4 mm	McMaster Carr	2958A34
Drill bit 7/64 in	McMaster Carr	2901A507
Slotted screwdriver bit 2mm, with 4mm hex shank	McMaster Carr	5750A375
Slotted screwdriver bit 1.5 mm, with 4mm hex shank	McMaster Carr	5750A373
Soldering iron	Grainger	https://www.grainger.com/product/WELLER-Solering-Station-1-Channel-799RP8
Silicone glue	Grainger	https://www.grainger.com/product/GE-Silicone-Sealant-Premium-Silicone-22N770
Lubricant oil	Grainger	https://www.grainger.com/product/3-IN-ONE-Machine-Oil-Multipurpose-Oil-41MW02
Solder	Grainger	https://www.grainger.com/product/AMERICAN-BEAUTY-Solder-Wire-1-32-in-x-11-g-19YP69
Plastic tip tweezers	DigiKey	2014-2ACFR.SA.1.ITU-ND
Pin connector male	DigiKey	350-10-101-00-006000
Socket connector female	DigiKey	2-5331272-3
Silver or Stainless Steel PFA coated wire	A-M Systems	786500 791600
Tough 2000 photopolymer resin	Formlabs	RS-CFG-TO20-01
Clear V4 photopolymer resin	Formlabs	RS-CFG-GPCL-04
Resin tank V2.1	Formlabs	RT-F3-02-01
Build platform	Formlabs	BP-F3-01
Form3 SLA 3D printer, wash, and cure	Formlabs	PKG-F3-SVC-COMPLETE
Neuropixels 1.0 probe	Neuropixels	PRB_1_4_0480_1
1.0 headstage and cable	Neuropixels	HS_1000, CBL_1000
PXIe card	Neuropixels	PXIE_1000
PXIe-1082 Chassis	National Instruments	PXIe-1082
A.M.P.I. Iso-flex Electrical stimulus isolator	A.M.P.I.	Iso-flex
A.M.P.I. Master-9 pulse stimulator	A.M.P.I.	Master-9
Metabond Kit	Parkell	S380
M1.4x12 screw (0.3 mm pitch)	McMaster Carr	91800A711
M1.4 x 4 screw	McMaster Carr	91430A156
M1 threaded insert	McMaster Carr	92120A110
M1.4 threaded insert	McMaster Carr	92120A140
M1 nut	McMaster Carr	90591A311
M2.6 x 25 screw	McMaster Carr	90353A120
M2.6 nut	McMaster Carr	90592A008
Drill bit 1.0 mm	McMaster Carr	2958A25
Drill bit 1.4 mm	McMaster Carr	2958A34
Drill bit 7/64 in	McMaster Carr	2901A507
Slotted screwdriver bit 2mm, with 4mm hex shank	McMaster Carr	5750A375
Slotted screwdriver bit 1.5 mm, with 4mm hex shank	McMaster Carr	5750A373
Soldering iron	Grainger	https://www.grainger.com/product/WELLER-Solering-Station-1-Channel-799RP8
Silicone glue	Grainger	https://www.grainger.com/product/GE-Silicone-Sealant-Premium-Silicone-22N770
Lubricant oil	Grainger	https://www.grainger.com/product/3-IN-ONE-Machine-Oil-Multipurpose-Oil-41MW02
Solder	Grainger	https://www.grainger.com/product/AMERICAN-BEAUTY-Solder-Wire-1-32-in-x-11-g-19YP69
Plastic tip tweezers	DigiKey	2014-2ACFR.SA.1.ITU-ND
Pin connector male	DigiKey	350-10-101-00-006000
Socket connector female	DigiKey	2-5331272-3
Silver or Stainless Steel PFA coated wire	A-M Systems	786500 791600
Tough 2000 photopolymer resin	Formlabs	RS-CFG-TO20-01
Clear V4 photopolymer resin	Formlabs	RS-CFG-GPCL-04
Resin tank V2.1	Formlabs	RT-F3-02-01
Build platform	Formlabs	BP-F3-01
Form3 SLA 3D printer, wash, and cure	Formlabs	PKG-F3-SVC-COMPLETE
Neuropixels 1.0 probe	Neuropixels	PRB_1_4_0480_1
1.0 headstage and cable	Neuropixels	HS_1000, CBL_1000
PXIe card	Neuropixels	PXIE_1000
PXIe-1082 Chassis	National Instruments	PXIe-1082
A.M.P.I. Iso-flex Electrical stimulus isolator	A.M.P.I.	Iso-flex
A.M.P.I. Master-9 pulse stimulator	A.M.P.I.	Master-9
Metabond Kit	Parkell	S380

### Experimental model and study participant details

The project received approval for all protocols from the University of Texas at El Paso Institutional Animal Care and Use Committee and followed the Guide for Care and Use of Laboratory Animals (IACUC reference number: A-202009-1). Sprague-Dawley rats (Charles River Laboratory Strain Code 001) were housed in ventilated cages under a 12:12 light/dark cycle at 22 ± 2°C and 50 ± 10% humidity with two rats of the same sex per cage before surgery, and single-housed in a custom cage after surgery (see Methods section *“Home cage modification”*). They had access to food and water *ad libitum* while in the home cage. Two male rats between 6 months and 2 years of age were used for this study and weighed 440 g and 550 g on average.

### Method details

#### Calculating vertical probe location

##### Distance as a function of drive screw turns

By dividing the rotations of the drive screw in the implant’s screw mechanism, one can control the distance that the shuttle travels along the shaft of the screw. If the amount of distance traveled per whole rotation is equal to the pitch of the drive screw, subdividing the rotations would divide the distance proportionally. Such granularity also slows the shuttle down significantly, which can prevent excessive stress on the probe shank as it moves through tissue. We can model the distance the shuttle would travel along the drive screw shaft as a function of turns at the drive screw.

(Equation 1) $$D=P\;T_{Dr}$$

Where:

∉ *D* is the distance in millimeters,

∉ *P* is the drive screw pitch,

∉ *T*_*Dr*_ is the direct turns of the drive screw.

For example, if one would wish to make the shuttle and probes travel 0.06 mm along the shaft of the drive screw by hand, we can figure out how many turns it would take to achieve this by solving for *T*_*Dr*_ if we know that the drive screw pitch is (0.3 mm):

$$T_{Dr}=\frac{D}{P}=\frac{0.06}{0.3}=0.2$$

Meaning that it takes 0.2 turns of the drive screw to make the shuttle travel 0.06 mm along the drive screw. This is not achieved easily when done by hand, in most cases.

To achieve these precise movements, we developed the “Kepler screwdriver” (or simply *“Kepler driver”* or *“Kepler”*), a mechanical screwdriver designed with cascading planetary gears, each with a gear ratio (GR) of 5:1, resulting in a total GR of 25:1 (see section *“Gear ratio calculations”*). This means that it takes 25 whole turns at the input of the system to complete 1 rotation at the output. With this GR, we can figure out the distance traversed by the shuttle along the shaft of the drive screw as a function of turns at the input of Kepler (*T*_*K*_) instead of turns at the drive screw directly:

(Equation 2) $$D=P\;\frac{T_{K}}{Gr}$$

Where:

∉ *D* is the distance in millimeters,

∉ *P* is the drive screw pitch,

∉ *T*_*K*_ is the turns at the input of Kepler,

and *Gr* is the gear ratio of Kepler.

Expanding upon the previous example, if we wish for the shuttle to travel the same 0.06 mm, we can solve for *T*_*K*_ if we know that the gear ratio of the Kepler driver is 25:1 (25/1) and the drive screw pitch is 0.3 mm:

$$T_{K}=\frac{D}{P}\;Gr=\left(\frac{0.06}{0.3}\right)\;\left(\frac{25}{1}\right)=5$$

This tells us that we can make the probe and shuttle travel 0.06 mm within the implant by turning Kepler only 5 times, which is much easier to do than the 0.2 turns of the drive screw that we calculated above.

##### Probe location during and after surgery

We can estimate where the tip of the probe is while the probes are inside the brain tissue through a series of calculations that can be made during and after surgery. During surgery, we first figure out the distance *L*_*0*_ the shanks must be drawn to reach a target implantation depth to have a known starting point. This is a function of the target initial implantation depth (*DV*_*0*_), skull thickness (*A*), and the implant’s thickness and the bottom-most wall (*B*):

(Equation 3) $$L_{0}=\left|DV_{0}\right|+A+B$$

Where:

∉ *L*_*0*_ is how far the shank must be drawn to achieve an initial implantation depth of *DV*_*0*_,

∉ *|DV*_*0*_*|* is the absolute value of the target initial implantation depth,

∉ *A* is the skull thickness, which is measured directly using calipers,

And *B* is the thickness of the implant wall at the bottom.

For example, to implant at a depth of *DV*_*0*_ = −1.4 mm on a rat with a skull thickness of *A* = 0.7 mm, and assuming the implant has a wall thickness of *B* = 0.5 mm, the shank must be drawn *L*_*0*_ *= |-1.4| + 0.7 + 0.5 = 2.6 mm.*

To then figure out how many *direct* drive screw turns it takes to draw the shank to this length, we must plug L_0_ from Equation 3 into Equation 1 as D and solve for *T*_*Dr*_.

$$T_{Dr}=\frac{D}{P}=\frac{2.6}{0.3}=8.67\;turns$$

We use a similar concept to know where the tip of the shank might be after each micro-adjustment, but instead of using Equation 1 to calculate the direct drive screw turns, we use Equation 2 to calculate the turns using the Kepler screwdriver since that gives us the most precise adjustment.

For example, if we want to make an adjustment of *D* = −0.06 mm into the brain tissue, we can plug the absolute value of *D* into Equation 2 and solve for *T*_*K*_. We know that the drive screw pitch *p* = 0.3 and that the gear ratio of Kepler is *GR* = 25/1, thus:

$$T_{K}=\frac{\left|D\right|}{P}\;Gr=\frac{0.06}{0.3}\;25=5\;turns$$

Finally, the approximate depth *DV* at which the tip of the probe shank is located within the brain after surgery is the initial implantation depth (*DV*_*0*_), plus the sum of each micro-adjustment distance (*D*_*i*_) made post-surgery.

(Equation 4) $$DV=DV_{0}+\sum_{n}^{1}Di$$

In this way, we can keep track of the shank depth several days after implantation and know how far we need to drive the probes into the brain to reach a target recording area. This information can then be used to calculate where a specific row of electrodes on the electrode array shank is located based on the electrode pad size and spacing between pads. We have created an Excel sheet when one may log each micro-adjustment to keep track of where the tip of the shank is over time and have made it available on GitHub (https://doi.org/10.5281/zenodo.16126335).

#### The Kepler screwdriver

The Kepler screwdriver, or simply “*Kepler driver*” *or “Kepler”* (Figure 1B) is a modular (Figure 3A), specialized screwdriver with a two-stage planetary gear system (Figures 3B–3E) encased within. It is compatible with any screwdriver bit with a 4 mm hexagonal shaft (Figure 3F). The gear mechanism divides the speed and amount of turns at the input knob by 25 thanks to its gear ratio (see “Gear ratio calculations”). This is to divide the turns of the implant’s drive screw to increase precision of any adjustments made to the vertical position of the probes, as well as minimize the possibility of human error when tuning the screw (see “Preventing miscounting of turns”). We provide a full build guide for the Kepler driver and all files related to the Kepler driver in our GitHub repository (https://doi.org/10.5281/zenodo.16126335).

##### Gear ratio calculations

In a gear-driven system, the proportion of output rotations to input rotations is called *turn ratio*, determined by the *gear ratio (GR*) of the system; herein, we will simply refer to the GR for simplicity. In the simplest spur gear arrangement, the GR of the system is determined by the number of teeth on the driver gear (input) and the teeth on the follower gear (output). The number of teeth on a gear also determines the diameter of the gear itself because since the two gears are expected to fit into each other, both the size of the teeth and the space between teeth must allow for the teeth on both gears to interlock. Therefore, if a driver gear has less teeth than the follower gear it will also be smaller and will complete several rotations before the follower manages to complete one, if the output of the system is placed on the follower gear, the input rotational movement at the driver gear will be slowed down at the output. However, a spur gear arrangement with large GR requires gears with many teeth and can thus become cumbersomely large and difficult to produce. Because the *Kepler driver* was meant to be portable and possibly used during rodent surgeries, we were constrained on the size of the device.

To make the mechanism compact, we decided on implementing a concentric, cascading planetary gear arrangement. This minimizes the amount of space that the gears take and facilitates 3D printing of the entire screwdriver using a commercially available SLA printer (resin printer) to cut down on the cost of material. The whole mechanism consists of two planetary gear assemblies where the output of one is transferred to the input of the other. Cascading planetary gears multiply the GR of each stage, resulting in a much higher GR in a compact form factor. It also avoids plane movement transferring or offsetting the output from the input drive; movement is transferred vertically directly down the center of the system to the output. A planetary gear is composed of an outer ring gear within which a carrier rests, and on the carrier are several planetary gears and a sun gear in the center.

In our design, the input to the mechanism is the sun gear and the output is the carrier, while the ring gear is held in place. For a setup like this, the inverse of the GR is given by the equation:

(Equation 5) $$\frac{1}{Gr}=\frac{S}{\;\left(R+S\right)}$$

We sought to achieve a GR of 25:1 because this would give us the smallest distance per turn that was useful for our purposes. This would then mean that 25 rotations at the input equals one rotation at the output, which when using a drive screw mechanism with a pitch (spacing between threads) of 0.3 mm, the smallest distance an object would travel along the shaft of the screw would be 0.012 mm (0.3 mm/25 = 0.012 mm). If we plug in our values for the gear teeth, we get the gear ratio for each stage of the screwdriver’s mechanism.

$$\frac{1}{Gr}=\frac{8}{\left(32+8\right)}=\frac{1}{5}$$

By cascading the two planetary gears, the GR of each stage is multiplied, resulting in a gear ratio of …

$$Gr=\left(\frac{1}{5\;}\right)\;\left(\frac{1}{5}\right)=\frac{1}{25}$$

Although implementing cascading planetary gears does increase the complexity of the device and the number of components to print, everything can still be printed over several 3D printing jobs with the same 1 L cartridge of resin. We have also provided an assembly guide and all the STL files for the screwdriver to guide users through the process of building the device (https://doi.org/10.5281/zenodo.16126335).

##### Preventing miscounting of turns

Making fractions of a turn easily visualizable prevents miscounting, especially after long surgeries. We designed an optional mechanical counter that can be attached to Kepler (Figure 3G). The counter simply transfers the input rotations to a 10:1 planetary gear (sun gear: 5 teeth; planet gear: 20 teeth; ring gear: 45 teeth) where the carrier is the output and the ring gear is fixed (Figures 3H and 3I). A disk which explicitly indicates whole, and half rotations is attached to the carrier, and thanks to the gear system, will only rotate a 10th of a turn per full rotation of the input knob. The rotational movement of the whole counter mechanism is directly transferred to the input of the first 5:1 gear stage of the screwdriver, so the input to the screwdriver goes unchanged. The knob for the counter has 0.25, 0.5, 0.75 indicators as a reference for the fractions of a rotation. The counter attaches to the device at the top via a modified version of the original enclosure which can be easily replaced. This thus continues the theme of modular design which the implant itself features.

#### Probe testing and calibration

##### The saline bath

To test probes that have already been loaded into an implant, we designed a saline bath (Figure 1C) which can be 3D printed and is adapted to our implant’s form factor. The bath features a tray to hold saline in, two copper plates which serve as stimulating electrodes which flank the probes on both sides connected to standard banana connectors, and small receptacles distal to the probes where the ground electrode(s) can be placed during testing. To hold the implant, we included a miniaturized holding block specific for the saline bath where the implant can rest while still allowing the shanks to be lowered into the saline. This block is separate from the bath (Figure 4F) to allow for adaptation to different implant shapes. When placed onto the bath, the block holds the implant securely above the saline. This setup allows for noise and gain testing on the probes as a troubleshooting step, or as a peace-of-mind step to take before implantation onto a live rat. Because the holding block is not built into the bath, one may remove it and test the probes inside the implant or by themselves without the implant under a stereotaxic device (Figures S2E and S2F). This can be achieved by holding onto the shuttle and probes using our shuttle holder (Figure S2F) or directly holding the probes using the manufacturer’s dovetail holder (https://www.neuropixels.org/probes-np1-0) if the probes have a metal cap. The saline bath and stereotaxic holders, as well as assembly instructions for the saline bath can be found on GitHub (https://doi.org/10.5281/zenodo.16126335).

##### Gain tests

Testing of the probes was done using our custom saline bath (Figure 1C) following the instructions in the Neuropixels 1.0 probe user manual *before* a probe was implanted. To test gain, a built implant with one fully retracted mounted probe was placed on the mini holder block (Figure 4F) with external reference and ground connected to an Iso-flex’s electrical stimulation isolator’s (see *“Materials list”* section *“Other hardware”*) ground terminal. The block was then slotted onto the saline back above two stimulating electrodes. The bath was filled with sterile saline (0.9 g NaCl per 100 mL PBS) and the two banana connectors (one red for the cathode and one black for the anode) were plugged into the stimulator. The probe’s Zero Insertion Force (ZIF) connector was mated with one headstage’s Flat Printed Circuit (FPC) connector and the headstage was plugged into a Neuropixels card on a PXIe-1082 Chassis. The stimulator was set to deliver a current of 260 μA and was connected to a Master-9 (see materials list section *“Other hardware”*) with a pulse train routine of 100, 100 μs square pulses, spaced 250 ms apart for triggering; this setup was left on standby until needed. After an acquisition was started through software (SpikeGLX, Open Ephys GUI, or Bonsai), the probe was lowered into the PBS by turning the drive screw clockwise until at least the first 3 mm of the probe shank were submerged; a change in the recorded signal from large to low-amplitude noise confirmed that the recording electrodes had been submerged. Nominal gain was set to 1000 on both AP and LFP channels via software. The Master-9 was then triggered to start the pulse train routine and spiking patterns matching in frequency to the pulse train were observed on the acquisition window with little background noise. The stimulation amperage was adjusted to avoid saturating the recording equipment if the positive and negative peaks of the signal were clipped. After measuring the recorded signal’s amplitude, we compared the recording to the true value set on the stimulator and calculated the gain by dividing the measured amplitude over the true amplitude. As indicated in the user manual, the resulting gain should not deviate more than 10% of the value set on the electrodes.

##### Noise tests

Noise tests were not performed because we did not have a faraday cage on hand, which would prevent us from appreciating the true noise characteristics of the system and bias our noise tests. Nevertheless, we deemed noise levels acceptable if we were able to observe the stimulation signal with the external reference placed in one of the ground receptacles located distal to the stimulating electrodes on the saline bath, or after changing the electrical reference of the probes from external to tip.

##### Implant calibration

The calibration block (Figures 4G and S1G) is used to calibrate an implant after assembly and before surgery. The following steps were taken to calibrate each implant used in this study: 1) The probe and shuttle were retracted to their highest position by turning the drive screw clockwise (cw). 2) The drive screw was then turned counterclockwise (ccw) 10 times for an initial probe displacement of 3 mm (see ‘calculating vertical probe location’). The tip of the probe shank was then compared against the 3 mm mark on the calibration block under a microscope. Because there exists a vertical tolerance built into the implant to safely accommodate the entirety of the probe shank inside the device, and because of small errors in the 3D printing process, the tip of the shank will most likely not align to the mark (Figure S2G). 3) This error distance (*ED*) was calculated by counting the number of ccw turns until the tip lined up with the center of the mark. The total ccw turns made up to this point were noted. 4) 10 turns were then subtracted from the total turns. The remaining turns (error turns, *ET*) were the number of turns needed to correct for *ED*. The ED was quantified using Equation 1*.* 5) The probe was returned to its fully retracted position, then the screw was turned *ET* times. This resulted in the tip lining up with the edge of the opening on the bottom of the skull connector (Figure S2H). 6) Finally, the screw was turned ccw 10 times again and the tip was compared against the mark on the calibration block; after this process, the tip aligned to the center of the mark signaling a successful calibration (Figure S2I). This process can be practiced safely using our practice probe (Figures 4H and 4I).

#### Surgical and post-surgical procedures

##### Tracking probe depth during and after surgery

To know the vertical location of the probes (*DV*), we first inserted the probe shanks at a known depth (*DV*_*0*_). To do this, we measured the skull thickness by directly measuring the bone extracted from the trephine hole using calipers. Although measuring with calipers is preferred to prevent damage to the probes, this distance may also be measured with a stereotaxic device by subtracting the DV at which the probe tips touch the bone at the edge of the trephine hole, from the DV at which the probes touch the brain tissue. The thickness of the bottom wall of the skull interface (typically 0.5 mm) is then added to the skull thickness (see ‘calculating vertical probe location’, Equation 3), and the probes are drawn out to this total distance by turning the drive screw counterclockwise the appropriate amount of times (determined by Equation 1). This length of the probe shank is then implanted into the brain. Any micro-adjustment (**see ‘Post-surgical micro-adjustments’**) made after surgery (*DV*_*1-n*_) is also tracked and cumulatively added to the initial implantation depth (Equation 4). For more information, see Methods S1. We have created an Excel sheet where one may log each micro-adjustment to keep track of where the tip of the shank is over time and have made it available on GitHub (https://doi.org/10.5281/zenodo.16126335).

##### Implantation surgery and post-surgical care

To ensure that the implanted probes are useful for as long as possible, several considerations must be considered. To start, the implant must be affixed to the rat’s head in the most stable way possible to prevent it from being torn off by the rat. To do this, we scored the skull and added various anchor screws around the implant (Figures 5A and 5B), then covered them in dental cement so that the whole implant had a strong foundation (Figure 5C). The screws must be driven deep enough into the bone that they are secure, but not deep enough to cause damage to the brain. This in combination with a large foundation of dental cement covering the entirety of the exposed bone keeps the implant in place after surgery (Figures 5D and 5E). After the probes have been introduced into the tissue, applying Vaseline around the “foot” of the implant where it contacts the skull can prevent discharge and blood from making its way into the body of the implant to prevent buildup from forming along the shuttle rails and essentially gluing the shuttle in place hindering further vertical adjustment of the probes. At the end of the surgery, we cauterized the remaining tissue around the implant with silver nitrate to prevent the tissue from regenerating under the dental cement and lifting the entire implant from its anchor. Post surgical care is also important in not only keeping the animal comfortable and healthy but also prevents implant failure. To prevent infections, provide pain relief and hydrate, animals were given routine broad-spectrum antibiotic injections (Enrofloxacin) and an injected NSAID (Meloxicam) in 10 mL of Ringer. Then, to reduce itching and locally disinfecting the surgical site, we routinely applied Chlorhexidine and a triple antibiotic on the live tissue around the implantation site. A combination of nystatin (antifungal), neomycin (antibiotic), thiostrepton (antimicrobial), and triamcinolone (steroid) was also used to treat this area. Hyaluronic acid can later be applied to keep the area moisturized after the tissue has scarred (see Methods S1 for additional information).

##### Home cage modification

Modifications to the animal’s home cage can help extend the lifespan of the implant by creating an environment that is comfortable to the rat with a large object attached to its head. A cage which is twice as tall as a standard rat home cage will allow the animal to move and rear freely. A taller cage necessitates giving access to water from the side of the cage instead of from the top. Additionally, reducing the length of the waterspout that protrudes into the cage helps eliminate points where the animal’s head can get stuck on which could lead to pulling on the implant until it rips off. The waterspout should not protrude more than 20 mm into the cage and should be situated close to the bottom of the cage or high enough to prevent the animal from getting stuck underneath it. For more information, see Methods S1.

#### Post-surgical micro-adjustments

Immediately after surgery, it is likely that there will be a very aggressive inflammation response in the tissue. Over time, scarring may occur around the electrode shank which may put the probes at risk of breaking if the probes are lowered too quickly after surgery. Scar tissue forming around the electrodes may also degrade the recorded signal over time. To combat the dangers of moving a fragile object through scarring, changing tissue and the natural degradation of the signal due to brain tissue immune response, we leveraged the adjustable nature of the movable shuttle by making a series of post-surgical micro-adjustments using our Kepler screwdriver. With this approach, we were able to slightly adjust the position of the probe shank within the brain tissue in a slow and controlled manner to avoid breaking the shank and keeping track of where the tip of the shank is after each adjustment (see ‘calculating vertical probe location’). We were able to reach our target at the Dorsal Striatum (−3.45 mm DV) within 3 weeks (after an initial implantation depth of −1.5 mm) by making 100–300 μm adjustments 2–3 times per week. Afterward, only 50 μm adjustments were made every other week or as soon as the recorded signal had degraded to a degree where spikes were undiscernible from the local field potentials (LFP) to maintain a relatively good SNR for at least 3 months after surgery.

#### Grounding considerations

##### Ground needs to be stable and quiet

Typically, electrical ground is needed to aid in the denoising of electrophysiological signals during the pre-amplification stage of the signal processing pipeline. Because voltages at the input of an amplifier are measured with respect to ground, ground must be as free of noise as possible to avoid unintentionally introducing artifact signals into the system, as this artifact will be present at the output of the amplifier. Additionally, grounding serves as a low-impedance path for current flow and acts as shielding against electromagnetic interference (EMI). For this reason, a quiet and stable grounding circuit must be present in any electrophysiological recording equipment, as the very low amplitude signals (roughly 10–100 μV) produced by neurons are extremely susceptible to large-amplitude noise and artifacts which may range from 1 mV to several volts, depending on the source. If no grounding is present at all, the entire recorded signal will at least be corrupted by 60 Hz line noise, and any other EMI present in the recording environment (for example, from the host computer) which impedes spike sorting later down the pipeline.

To ground the probes within the implant, we included a simple grounding circuit within the implant, which extends out to a small copper plate through which a screw is placed. This not only connects the probes and screw together but also makes it so that the surgeon may place the ground on whichever screw on the skull. We chose copper for this plate because unlike stainless steel, which is what screws tend to be made of, copper is a low-impedance metal and takes solder well, which is important for affixing the ground wire. Any other similarly conductive material would be appropriate (such as a tin-copper alloy) if it solders well. For the wire, a decently conductive material with low impedance such as Silver should be chosen to allow current to pass onto the ground, however, it is important to note that the total impedance of the ground circuit will be equal to the summation of resistances of each element of the grounding circuit, provided that only resistive elements such as the wire, plate, and screw exist in the circuit and no other capacitive or inductive elements are introduced. Maintaining a clean, simple, low-impedance ground will aid in keeping recorded signals as free of artifacts as possible.

##### Avoiding muscle tissue on ground circuit

Because muscle tissue is electrically active, it is common for it to be a prominent source of artifact in electrophysiological recordings when it meets any part of an electrical circuit. For electrode arrays, the reference, or particularly, the grounding circuits are the most susceptible as they tend to be outside of the tissue they are recording from and may contact electrically active tissue, particularly muscle. Voltage readings from muscle activity result in signals that are magnitudes larger in amplitude than extracellular neuronal signals and may contain similar frequency components. This means that a recording from brain tissue can easily be obscured by electromyographic signal patterns both in the voltage and frequency domains, which can complicate filtering, hinder spike detection, and prevent accurate spike sorting later down the pipeline.

There are a variety of muscles located on the rat skull. Of special importance, however, is the temporalis muscle, due to its location. Most procedures for brain electrophysiology implants, including ours, require reflecting the temporalis muscle at the dorsal crest of the rat skull to access the bone and perform a craniotomy to implant the probe shanks into. This large muscle extends beyond this area however, and is involved in jaw closing, which means it will be very active throughout any recording session as a rat bruxes, grooms, or eats. Avoiding electrical contact with this muscle is crucial for clean recordings that are useful for spike sorting algorithms.

To avoid contacting muscle with any component of the grounding circuit on the probes, we create a large incision on the rat’s head which extends from between the eyes to just behind the ears. We then reflect the skin and muscle from the implantation site and scrape the connective tissue off of the skull to make enough space for grounding screws and ensure they are not contacting any surrounding tissue. We also coat the screws in dental cement to shield them from the tissue. This prevents the ground screws from contacting any surrounding muscle and provides a sufficiently “quiet” ground for the probes, which prevents (or at least partially prevents) electromyographic artifacts from contaminating our recordings.

##### Avoiding ground loops

Having multiple recording devices (probes) on the same rat may present a challenge with grounding. Avoiding ground loops is extremely important so that each probe has a quality reference point from which to measure voltage from, which in turn prevents the recorded signal from being corrupted by EMI. Ground loops occur when 2 or more devices are interconnected through their ground circuits. The resulting ground circuit creates a physical closed loop, which acts as a single-turn secondary winding of a transformer with the primary winding being the summation of all the magnetic fields produced by neighboring devices. When oscillating (50–60 Hz) ambient magnetic fields pass through the ground loop, an oscillating current is induced and conducted through the grounding wire, into the recording device, and consequently introducing EMI into the recorded signal. Because of the low resistance of the wire, even a small ambient magnetic field can induce a current in the ground loop which can significantly interfere with the low-amplitude recordings. In audio equipment, the induced EMI can present itself as a constant low-frequency hum.

To prevent ground loops when two probes are present on the implant, it’s important to give each probe its own separate ground circuit. One can achieve this by simply avoiding interconnection of each probe’s ground screw during surgery. If more than one screw is included in a probe’s ground circuit, the same applies; that probe’s ground circuit must not be connected to the other probe’s ground. In a single probe setup, limit the number of ground screws in the circuit. Each probe, whether in a single or double probe configuration, should have at minimum one ground screw placed in an area distal to the recording site, and away from any tissue other than bone. Accidental ground loops, where the same wire comes into contact with itself, are also prevented by using wire that has been coated with an electrically insulating material such as Perfluoroalkoxy (PFA). Additionally, as mentioned in a previous section, minimizing the surface area that the ground circuit covers as long stretches of (un-looped) wire can also act as an antenna and pick up EMI. The ubiquitous nature of ambient magnetic fields makes avoiding ground loops an extremely important step in denoising a recorded signal; although EMI can be filtered away, it is always best to avoid picking it up altogether to get the cleanest, most artifact-free signal possible.

#### Recording and signal processing

##### Recording environment and procedure

Rats were anesthetized prior to recording using vaporized Isoflurane in order to connect the headstage safely, then individually recorded freely moving in a dark room from within their home cages with the lid removed. Rats were supervised but with minimal interaction between the rat and the experimenter. The data acquisition equipment used was a PXIe-1082 chassis with a Neuropixels PCIe card recording from one Neuropixels headstage and Neuropixels 1.0 probe on each rat. Recordings (50 min each) were made on days 75, 76, 81, and 82 after surgery, with −60 μm adjustments made on days 74 and 81.

##### Signal pre-processing

Recorded signals were pre-processed and visualized in MATLAB using a combination of custom code and plugins (Figure 6A). The signal processing chain consisted of three ordered processing stages: 1) artifact identification and removal using visual inspection and thresholding (*Z* score = 10 threshold; defined as a number of standard deviations from the entire background signal on the processed channel), 2) common local field potential (LFP) averaging (referencing 7 channels above and/or below the processed channel), and 3) band-pass filtering (with 2^nd^ order Butterworth filter, filter pass-band = 300 : 5000 Hz). Source code for this signal processing chain can be found on GitHub (https://doi.org/10.5281/zenodo.16288405, under “Porting Open Ephys”).

##### Spike detection

Spike detection and alignment was done offline in MATLAB over one channel at a time after pre-processing. Spikes were detected with a threshold of −80 μV, then further filtered with a threshold of *Z* score = 1. The resulting traces were inverted and cut to 1.7 millisecond clips with maximum value of the spike as the median data point (Figures 6B and S4A). Detected spikes were aligned afterward by plotting each spike centered at the maximum value of each (Figures 6C and S4B). To relate the detected spikes to activity on neighboring channels, the timestamp of the highest-amplitude sample from each detected spike was saved and analyzed across the nearest 4 channels above and below (corresponding to neighboring electrodes). The same spike detection algorithm was run over these channels to find similar spiking activity in those channels (Figures 6D and S4C). This process was repeated over four days' worth of recordings to assess recording stability 2 days before and after an adjustment procedure (Figure S4D). Source code for this procedure can be found on GitHub (https://doi.org/10.5281/zenodo.16288405, under “Porting Open Ephys”).

#### Implant modifiability

##### Modularity facilitates modifiability

We have chosen a modular implant design so that any adaptations to the implant can be made in as little time as possible by isolating only key parts of the implant instead of always making modifications to the general structure. For example, although our ‘base’ implant is designed around the Neuropixels 1.0 probe, the implant can be adapted to a 2.0 probe in under a day by modifying only the shuttle. Assuming the general dimensions of the shuttle are left unchanged, the modified shuttle can still fit in the implant body and be driven by the same drive screw mechanism. If desired, the implant can be shortened to accommodate the smaller 2.0 probes in an additional day or two, only adapting two additional implant pieces (see ***‘Neuropixels 2.0 adaptation’***). In a similar fashion, individual implant parts can be modified to add stimulation electrodes and wires (see ***‘Electrical stimulation modifications’***), or optical fiber cannulas to include optogenetic stimulation capabilities on-board the implant (see ***‘Optogenetic modifications’***). Modified versions of the implant can be found in our GitHub repository (https://doi.org/10.5281/zenodo.16126335).

##### Mesh-oriented implant editing

The open-source, 3D software *Blender* (version 3.4.0) was chosen over CAD software for the development of this project to provide an alternative method for modifying the base implant and in this way allowing for more flexibility. Blender’s mesh-oriented 3D modeling makes modifications easy by enabling the user to modify individual sections on a mesh (*vertices, edges, or faces),* or by using non-destructive mesh editing through *Modifiers.* For example, if one wishes to alter the dimensions of the implant, the mesh can be scaled, or groups of vertices on the mesh can be moved around on the X, Y, or Z axes (see ***‘Neuropixels 2.0 adaptation’***). On the other hand, with an *Additive Boolean modifier*, an attachment can be 3D modeled and then attached to the base implant mesh (see ***‘Electrical stimulation modifications’***), or with a *Subtractive Boolean modifier,* holes and channels can be made on the implant to embed or made room for wires or optical fiber (see ***‘Optogenetic modifications’***). These modifications can also be made in CAD software such as AutoCAD, SolidWorks, or the open-source FreeCAD by modifying the STL files directly, but the inclusion of Blender as a 3D model-editing platform extends the modifiability of our implant to one additional platform and give the most power to the end-user.

##### Neuropixels 2.0 adaptation

Our implant can be easily adapted to the newer Neuropixels 2.0 probes by making quick and small modifications. As mentioned before, the shuttle is the main implant piece that needs modification to fit the 2.0 probe snugly on the implant. However, in this paper we have made modifications to the whole implant to provide a ready-made implant for the newer generation probes (Figure 7A, modified implant pictured left). The first modification made was to the shuttle, where it was shortened, and the *probe bed* was modified to fit the new probe’s form factor (Figure S5A, pictured left). The next modification was on the *implant body.* The body was shortened slightly, and the drive screw rail was elongated to ensure that the shuttle is fully protected regardless of where it is located on the implant (Figure S5B, pictured left). Finally, the skull interface was also shortened, and the *rail cover* was modified to fit the new screw rail on the body (Figure S5C, pictured left). In both the original and modified designs, the probe and shank are protected fully when the shuttle is at its highest position (Figures 7B and S5D). The result of this modification yielded a smaller implant, measuring approximately 3.6 cm in height compared to the 4.4 cm of the original implant (including the headstage interface and cap pieces). These three modifications were made over the course of two days and illustrate how the implant can be quickly modified to different probe form factors instead of needing to design an entirely new implant around a different probe which could take several weeks.

##### Electrical stimulation modification

We have provided an example modification to the implant that includes a connector for adding electrical stimulation capabilities to the implant (Figure 7C, pictured left). In this modification, the *implant body* is fit with a header pin receptacle (Figure 7D, pictured left) and stimulation wire connectors (Figure S5E). and the skull interface is modified so that 0.2 mm wire can stick out of the bottom of the implant (Figure 7E). The setup allows for the header pins and wires to be attached from the top of the implant, then the wires are fed into the skull interface via the side holes and finally soldered to stimulation electrodes affixed to the bottom. In this example, only the implant body and skull interface are changed while every other part of the implant fits in place without any modifications.

##### Optogenetic modification

The last modification to our implant that we made is one that allows for optical fibers and cannulas to be added to the build for experiments that include optogenetic stimulation. In this design, optical fibers come out of the bottom of the skull interface 1.25 mm away from each probe shank, 0.5 mm above the tip of the shank (Figure 7F*,* pictured left). This is achieved through the addition of receptacles where cannulas (1.1 mm diameter cannula for 0.1 mm diameter optical fiber) are affixed (Figure S5F). While the implant body and shuttle remain unchanged, the headstage interface is modified so that an additional two cannulas can be affixed on the top to create a receptacle to connect optical stimulators (Figure S5G). The top receptacles are offset and slightly tilted to move the optical fiber out of the way of the probe ribbon when it is folded inside the implant body.

#### Inflammation assessment

##### Implant preparation

Two of our base implants were printed, assembled, and calibrated in the same fashion described in this manuscript, each encasing one Neuropixels 1.0 probe. Probes and implants were sterilized in 70% isopropyl alcohol (IPA) prior to surgery.

##### Implantation and adjustment protocol

To visualize the foreign body inflammation response in the brain tissue using the methods described in this manuscript, one rat was implanted and immunostaining was done following a 16-day gradual adjustment protocol (Figure S6). On the day of the surgery, the probe was implanted at an initial implantation depth of −3.3 mm (see Methods ‘surgical and post-surgical procedures’). The adjustment protocol started the day after surgery and was done in three phases to reach the target depth of DV = −5.0 mm. For the first phase, we repositioned the probe by −48 μm on post-surgical days 1, 2, and 5–9; no adjustments were made on days 3 and 4. In the second phase, −300 μm adjustments were done on days 12–15, and in the third phase, a final −39 μm adjustment on day 16. All adjustments were done using the Kepler screwdriver at roughly 12 rpm (5 s per turn at input). The rat was perfused, and the brain was harvested 7 days after the final adjustment (23 days after implantation).

##### Euthanasia and tissue preparation

Animals were deeply anesthetized using isoflurane then transcardially perfused with 0.9% saline followed by 4% paraformaldehyde (PFA) in phosphate-buffered saline (PBS). The probes were then slowly retracted by turning the drive screw clockwise fully and the implants were removed. Finally, brains were extracted, post-fixed overnight in 4% PFA at 4°C, and transferred to a 30% sucrose solution in PBS for cryoprotection the next day for 48 h. Tissue slicing was done using a sliding microtome in 30 μm coronal sections which were then stored in cryoprotectant at −20°C until they were processed.

##### CD11b+c staining

Immunofluorescent detection of microglial-associated inflammation was done on striatal tissue containing the implant track. Sections were rinsed 5 times on an orbital shaker at room temperature in 1× Tris-buffered saline (TBS) for 5 min. The tissue was then incubated in blocking solution (1× TBS, 0.1% Triton X-100, 4% normal donkey serum) for 2 h at room temperature.

Primary incubation was done on cut sections for 48 h at 4°C using a mouse monoclonal anti-CD11b+c antibody (Abcam, ab1211; 1:500) diluted in blocking solution. Prior to secondary incubation, sections were rinsed in TBS (5 × 5 min), then incubated for 5 h at room temperature with A546 conjugated donkey anti-mouse IgG (Invitrogen A32787TR; 1:2000). Sections were rinsed again following secondary incubation.

For cryoarchitectural visualization, tissue was counterstained for 10 min using NeuroTrace 435/455 (Invitrogen N21479; 1:500 in TBS), followed by five final rinses in TBS. Finally, sections were mounted onto Superfrost Plus microscope slides, cover slipped with sodium bicarbonate-buffered glycerol, and stored at 4°C in the dark until imaging.

### Quantification and statistical analysis

Quantification and visualization of electrophysiological data was done using a custom algorithm which pre-processed raw data (filtering and artifact removal), performed spike detection via thresholding (−80 μV). The code has been made available on GitHub (https://doi.org/10.5281/zenodo.16288405, under “Porting Open Ephys”). This manuscript focused on implant development including 3D printing and engineering practices. There was no formal statistical analysis performed for the data presented herein.

### Additional resources

#### Repositories

• Repository containing all 3D printing files (STL and Blender) and assembly instructions.
  ○ https://doi.org/10.5281/zenodo.16126335.
• Repository containing scripts used for pre-processing and spike detection (MATLAB).
  ○ https://doi.org/10.5281/zenodo.16288405, under “Porting Open Ephys”.

## Supplemental information

Document S1. Figures S1–S6 and Methods S1

Document S2. Article plus supplemental information

## References

[1] Steinmetz N.A., Zatka-Haas P., Carandini M., Harris K.D. (2019). Distributed coding of choice, action and engagement across the mouse brain. Nature.

[2] Miller K.J., Botvinick M.M., Brody C.D. (2022). Value representations in the rodent orbitofrontal cortex drive learning, not choice. eLife.

[3] Liu K., Zhang H., Hu M., Li Z., Xu K., Chen D., Cui W., Lv C., Ding R., Geng X., Wei S. (2024). The past, present, and future of *in vivo* -implantable recording microelectrodes: the neural interfaces. Mater. Adv..

[4] Hong G., Lieber C.M. (2019). Novel electrode technologies for neural recordings. Nat. Rev. Neurosci..

[5] Strumwasser F. (1958). Long-Term Recording from Single Neurons in Brain of Unrestrained Mammals. Science.

[6] Muniak M.A., Mayko Z.M., Ryugo D.K., Portfors C.V. (2012). Preparation of an Awake Mouse for Recording Neural Responses and Injecting Tracers. J. Vis. Exp..

[7] Ledo A., Lourenço C.F., Laranjinha J., Gerhardt G.A., Barbosa R.M. (2018). Concurrent measurements of neurochemical and electrophysiological activity with microelectrode arrays: New perspectives for constant potential amperometry. Curr. Opin. Electrochem..

[8] Spira M.E., Hai A. (2013). Multi-electrode array technologies for neuroscience and cardiology. Nat. Nanotechnol..

[9] Wang C., Stratton P.G., Sah P., Marek R. (2023). A protocol to investigate neural coupling of brain oscillations in rodents using in vivo electrophysiological recordings. STAR Protoc..

[10] Lu L., Popeney B., Dickman J.D., Angelaki D.E. (2018). Construction of an Improved Multi-Tetrode Hyperdrive for Large-Scale Neural Recording in Behaving Rats. J. Vis. Exp..

[11] Dufour S., De Koninck Y. (2015). Optrodes for combined optogenetics and electrophysiology in live animals. Neurophotonics.

[12] Kim E.H., Chin G., Rong G., Poskanzer K.E., Clark H.A. (2018). Optical Probes for Neurobiological Sensing and Imaging. Acc. Chem. Res..

[13] Pendry R.J., Quigley L.D., Volk L.J., Pfeiffer B.E. (2023). A Lightweight Drive Implant for Chronic Tetrode Recordings in Juvenile Mice. J. Vis. Exp..

[14] Kloosterman F., Davidson T.J., Gomperts S.N., Layton S.P., Hale G., Nguyen D.P., Wilson M.A. (2009). Micro-drive array for chronic in vivo recording: drive fabrication. J. Vis. Exp..

[15] Yamamoto J., Wilson M.A. (2008). Large-Scale Chronically Implantable Precision Motorized Microdrive Array for Freely Behaving Animals. J. Neurophysiol..

[16] Voigts J., Siegle J.H., Pritchett D.L., Moore C.I. (2013). The flexDrive: an ultra-light implant for optical control and highly parallel chronic recording of neuronal ensembles in freely moving mice. Front. Syst. Neurosci..

[17] Voigts J., Newman J.P., Wilson M.A., Harnett M.T. (2020). An easy-to-assemble, robust, and lightweight drive implant for chronic tetrode recordings in freely moving animals. J. Neural. Eng..

[18] Guardamagna M., Eichler R., Pedrosa R., Aarts A., Meyer A.F., Battaglia F.P. (2022). The Hybrid Drive: a chronic implant device combining tetrode arrays with silicon probes for layer-resolved ensemble electrophysiology in freely moving mice. J. Neural. Eng..

[19] Fee M.S., Leonardo A. (2001). Miniature motorized microdrive and commutator system for chronic neural recording in small animals. J. Neurosci. Methods.

[20] Haumesser J.K., Kühn J., Güttler C., Nguyen D.-H., Beck M.H., Kühn A.A., Van Riesen C. (2017). Acute In Vivo Electrophysiological Recordings of Local Field Potentials and Multi-unit Activity from the Hyperdirect Pathway in Anesthetized Rats. J. Vis. Exp..

[21] Carter M., Shieh J. (2015). Guide to Research Techniques in Neuroscience.

[22] Bimbard C., Takács F., Catarino J.A., Fabre J.M.J., Gupta S., Lenzi S.C., Melin M.D., O’Neill N., Orsolic I., Robacha M. (2025). An adaptable, reusable, and light implant for chronic Neuropixels probes. eLife.

[23] Gregory B.A., Thompson C.H., Salatino J.W., Railing M.J., Zimmerman A.F., Gupta B., Williams K., Beatty J.A., Cox C.L., Purcell E.K. (2023). Structural and functional changes of deep layer pyramidal neurons surrounding microelectrode arrays implanted in rat motor cortex. Acta Biomater..

[24] Kozai T.D.Y., Jaquins-Gerstl A.S., Vazquez A.L., Michael A.C., Cui X.T. (2015). Brain Tissue Responses to Neural Implants Impact Signal Sensitivity and Intervention Strategies. ACS Chem. Neurosci..

[25] Sharon A., Jankowski M.M., Shmoel N., Erez H., Spira M.E. (2021). Inflammatory Foreign Body Response Induced by Neuro-Implants in Rat Cortices Depleted of Resident Microglia by a CSF1R Inhibitor and Its Implications. Front. Neurosci..

[26] Sahyouni R., Chang D.T., Moshtaghi O., Mahmoodi A., Djalilian H.R., Lin H.W. (2017). Functional and Histological Effects of Chronic Neural Electrode Implantation. Laryngoscope Investig. Otolaryngol..

[27] Steinmetz N.A., Koch C., Harris K.D., Carandini M. (2018). Challenges and opportunities for large-scale electrophysiology with Neuropixels probes. Curr. Opin. Neurobiol..

[28] Otte E., Vlachos A., Asplund M. (2022). Engineering strategies towards overcoming bleeding and glial scar formation around neural probes. Cell Tissue Res..

[29] Schröder T., Taylor R., Abd El Hay M., Nemri A., França A., Battaglia F., Tiesinga P., Havenith M.N., Schölvinck M.L. (2024). The DREAM Implant: A Lightweight, Modular, and Cost-Effective Implant System for Chronic Electrophysiology in Head-fixed and Freely Behaving Mice. J. Vis. Exp..

[30] Malekoshoaraie M.H., Wu B., Krahe D.D., Ahmed Z., Pupa S., Jain V., Cui X.T., Chamanzar M. (2024). Fully flexible implantable neural probes for electrophysiology recording and controlled neurochemical modulation. Microsyst. Nanoeng..

[31] Wang S., Li L., Zhang S., Jiang Q., Li P., Wang C., Xiao R., Li X.-M., Song J. (2024). Multifunctional ultraflexible neural probe for wireless optogenetics and electrophysiology. Giant.

[32] Fiáth R., Márton A.L., Mátyás F., Pinke D., Márton G., Tóth K., Ulbert I. (2019). Slow insertion of silicon probes improves the quality of acute neuronal recordings. Sci. Rep..

[33] Chen P.-C., Young C.G., Schaffer C.B., Lal A. (2022). Ultrasonically actuated neural probes for reduced trauma and inflammation in mouse brain. Microsyst. Nanoeng..

[34] Juavinett A.L., Bekheet G., Churchland A.K. (2019). Chronically implanted Neuropixels probes enable high-yield recordings in freely moving mice. eLife.

[35] van Daal R.J.J., Aydin Ç., Michon F., Aarts A.A.A., Kraft M., Kloosterman F., Haesler S. (2021). Implantation of Neuropixels probes for chronic recording of neuronal activity in freely behaving mice and rats. Nat. Protoc..

[36] Vöröslakos M., Petersen P.C., Vöröslakos B., Buzsáki G. (2021). Metal microdrive and head cap system for silicon probe recovery in freely moving rodent. eLife.

[37] Jun J.J., Steinmetz N.A., Siegle J.H., Denman D.J., Bauza M., Barbarits B., Lee A.K., Anastassiou C.A., Andrei A., Aydın Ç. (2017). Fully integrated silicon probes for high-density recording of neural activity. Nature.

[38] Steinmetz N.A., Aydin C., Lebedeva A., Okun M., Pachitariu M., Bauza M., Beau M., Bhagat J., Böhm C., Broux M. (2021). Neuropixels 2.0: A miniaturized high-density probe for stable, long-term brain recordings. Science.

[39] Putzeys J., Raducanu B.C., Carton A., De Ceulaer J., Karsh B., Siegle J.H., Van Helleputte N., Harris T.D., Dutta B., Musa S., Mora Lopez C. (2019). Neuropixels Data-Acquisition System: A Scalable Platform for Parallel Recording of 10 000+ Electrophysiological Signals. IEEE Trans. Biomed. Circuits Syst..

[40] Newman J.P., Zhang J., Cuevas-López A., Miller N.J., Honda T., van der Goes M.-S.H., Leighton A.H., Carvalho F., Lopes G., Lakunina A. (2025). ONIX: a unified open-source platform for multimodal neural recording and perturbation during naturalistic behavior. Nat. Methods.

[41] Stice P., Muthuswamy J. (2009). Assessment of gliosis around moveable implants in the brain. J. Neural. Eng..

[42] Rezayat E., Shayanfar F., HajiNasrollah M., Shakrian F., Dehaqani M.R.A. (2024). Custom-made Implants for Chronic In Vivo Electrophysiological Recording From Primate’s Brain Based on the Reconstructed Skull Model. Basic Clin. Neurosci..

[43] Lanz F., Lanz X., Scherly A., Moret V., Gaillard A., Gruner P., Hoogewoud H.-M., Belhaj-Saif A., Loquet G., Rouiller E.M. (2013). Refined methodology for implantation of a head fixation device and chronic recording chambers in non-human primates. J. Neurosci. Methods.

[44] Feingold J., Desrochers T.M., Fujii N., Harlan R., Tierney P.L., Shimazu H., Amemori K.I., Graybiel A.M. (2012). A system for recording neural activity chronically and simultaneously from multiple cortical and subcortical regions in nonhuman primates. J. Neurophysiol..

[45] Schwerdt H.N., Shimazu H., Amemori K.I., Amemori S., Tierney P.L., Gibson D.J., Hong S., Yoshida T., Langer R., Cima M.J., Graybiel A.M. (2017). Long-term dopamine neurochemical monitoring in primates. Proc. Natl. Acad. Sci..

[46] Horan M., Regester D., Mazuski C., Jahans-Price T., Bailey S., Thompson E., Slonina Z., Plattner V., Menichini E., Toksöz I. (2024). Repix: reliable, reusable, versatile chronic Neuropixels implants using minimal components. bioRxiv.

[47] Salatino J.W., Ludwig K.A., Kozai T.D.Y., Purcell E.K. (2017). Glial responses to implanted electrodes in the brain. Nat. Biomed. Eng..

[48] Nolta N.F., Christensen M.B., Crane P.D., Skousen J.L., Tresco P.A. (2015). BBB leakage, astrogliosis, and tissue loss correlate with silicon microelectrode array recording performance. Biomaterials.

[49] Franks W., Schenker I., Schmutz P., Hierlemann A. (2005). Impedance characterization and modeling of electrodes for biomedical applications. IEEE Trans. Biomed. Eng..

