# Supplementary material for: A modular, adaptable, and accessible implant kit for chronic electrophysiological recordings in rats
Source: Cell Rep Methods. 2025 Sep 2;5(9):101146. doi: 10.1016/j.crmeth.2025.101146 (PMC12539253; doi:10.1016/j.crmeth.2025.101146)
Supplement: Document S1. Figures S1–S6 and Methods S1 [file mmc1.pdf]

Cell Reports Methods, Volume *5*

Supplemental information

A modular, adaptable, and accessible implant kit for chronic electrophysiological recordings in rats

Raquel J. Ibáñez Alcalá, Andrea Y. Macias, Cory N. Heaton, Ricardo Sosa Jurado, Alexis A. Salcido, Neftali F. Reyes, Serina A. Batson, Luis D. Davila, Dirk W. Beck, Lara I. Rakocevic, Atanu Giri, Kenichiro Negishi, Sabrina M. Drammis, Ki A. Goosens, Travis M. Moschak, and Alexander Friedman

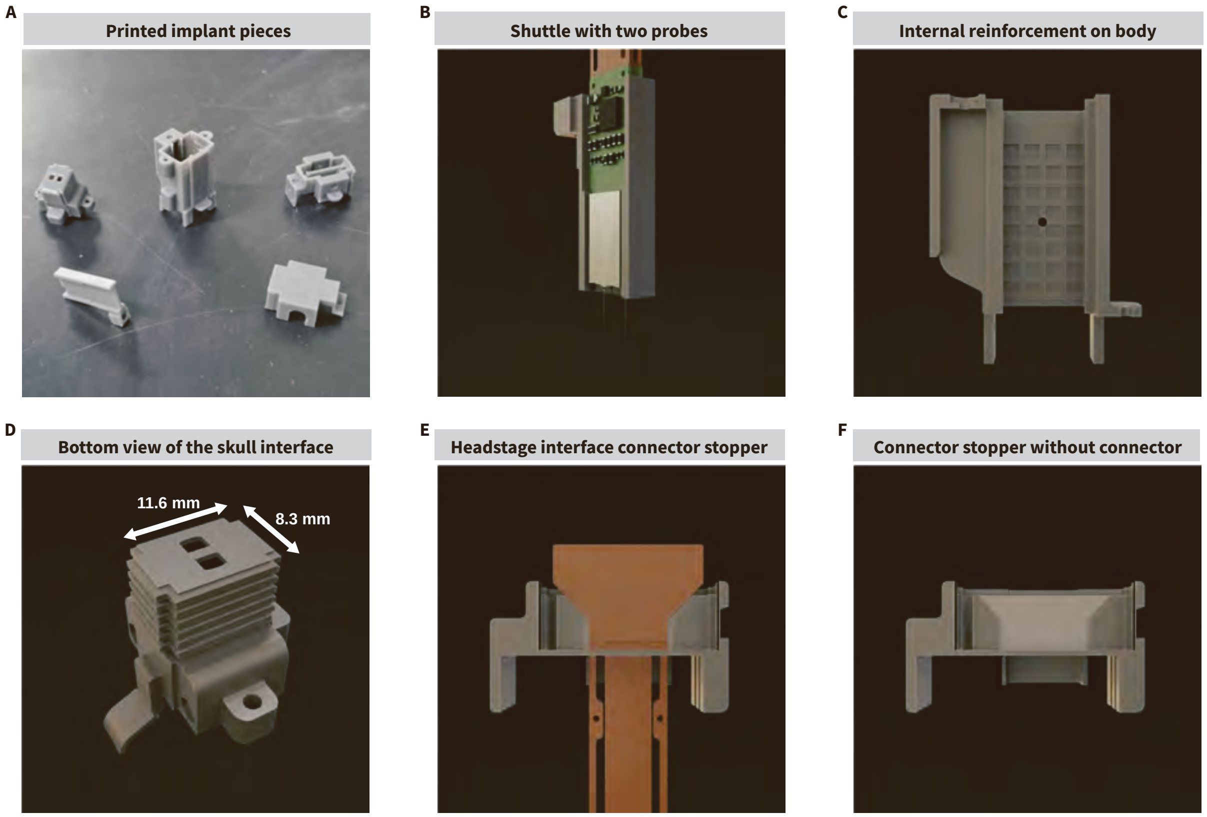

Figure S1. Implant components and design choices, related to **Figure 2. (A)** Photograph showing the individual 3D printed components of the implant. **(B)** The shuttle is designed with two "probe beds" on either side. Probes sit on the shuttle back-to-back and 3 mm apart. **(C)** Internal reinforcement of the implant body. The "waffle pattern" prevents the walls of the implant body from being pushed inward or breaking. **(D)** Bottom side of the skull interface showcasing the two holes where the probe shanks protrude out of the implant. The ribbed pattern on the skull interface helps increase the surface area which contacts the dental cement to strengthen the bond between it and the implant. **(E, F)** Bisected view of the headstage interface to showcase the ribbon stopper. This helps keep the probe's ribbon connector in place to facilitate connection of the headstage(s).

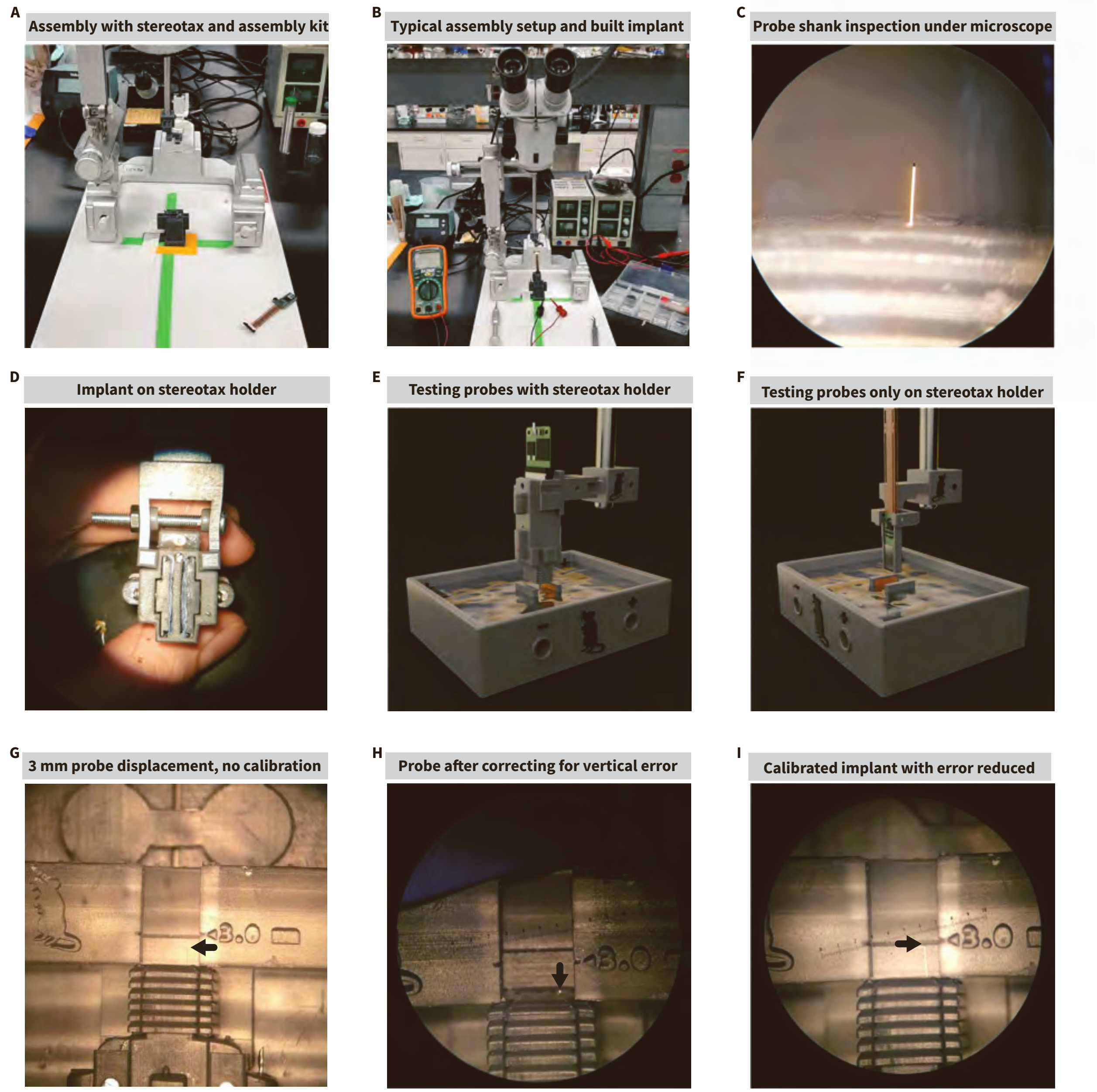

Figure S2. Assembly and testing setup, related to **STAR Methods (A)** Use of a stereotaxic device, shuttle holder adapter, and holder block in the assembly process. The shuttle adapter is placed on the stereotaxic device and the shuttle is mounted onto it. The skull interface piece is placed on the holder block underneath, and the shuttle is aligned and lowered into the skull interface. **(B)** Typical setup used to assemble the implant. A surgical microscope is used to monitor the probe shank(s) when lowering the shuttle into the skull interface piece. **(C)** Inspecting the probe shank under the microscope after assembling the implant. **(D)** Mounting the implant onto the stereotaxic holder implant adapter. **(E, F)** Alternative probe testing setups. **(E)** Instead of using the mini holder block, the implant is mounted onto the implant adapter and lowered into saline in the saline bath. **(F)** The probes are tested on the shuttle without the implant using the stereotaxic holder shuttle adapter. **(G - I)** Shank tip position marked by the black arrows prior to (G), during (H), and after calibration (I), viewed under a microscope. Before calibration, it is expected that the tip of the probe shank will not reach the 3 mm mark after 10 drive screw turns starting from a fully retracted position due to the built-in vertical tolerance and printing errors (G). After calculating the error distance, the tip is homed to a position where it is lined up with the threshold of the skull connector opening (H). From this position, the screw is turned 10 times and, if calibration is successful, the tip should line up with the 3 mm mark (I). The total calculated error for the implant pictured was 0.9 mm (3 drive screw turns).

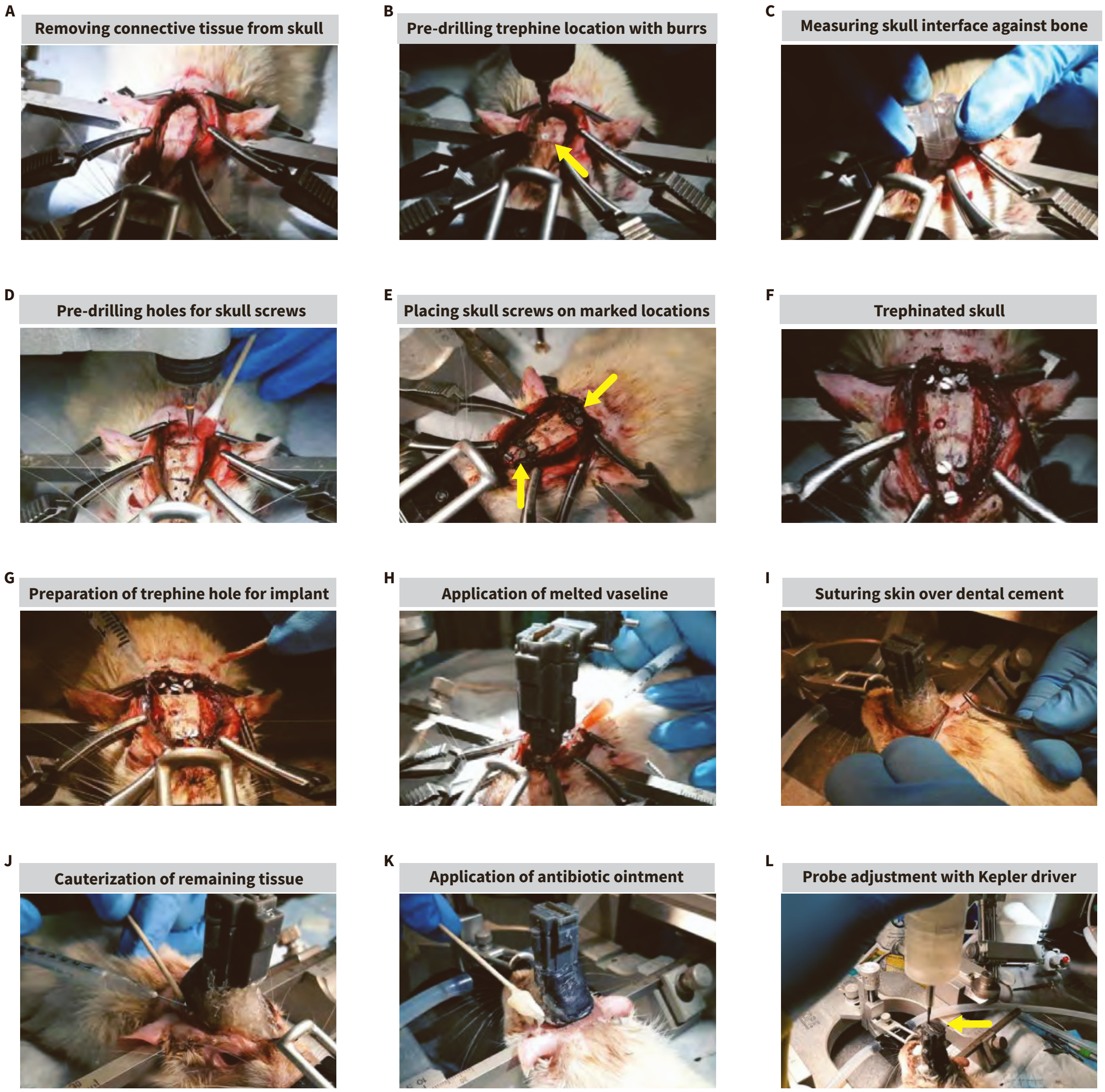

Figure S3. Additional surgical procedures, related to **Figure 5. (A)** Clearing the implantation site of connective tissue. **(B)** Marking the trephination site with a burrs drill bit. **(C)** Measuring and marking the edges of the skull interface piece to plan for anchor and ground skull screws. **(D)** Pre-drilling screw sites with a burrs drill. **(E)** Final placement of all skull screws. **(F)** Trephined skull. **(G)** Cleaning and hydrating the trephine hole with 4 µg/mL of Dexamethasone. **(H)** Application of melted vaseline to the edges of the implant after lowering it and inserting the probe shanks into the tissue. This helps prevent blood and discharge from seeping into the implant and prevents the shuttle from moving. **(I)** Suturing skin over the dental cement to prevent it from scarring underneath and lifting the implant over time. **(J)** Cauterizing the remaining tissue around the implant to prevent it from scarring under the implant. **(K)** Applying antifungal, corticosteroid, and antiseptic ointment on the exposed tissue to aid the healing process. **(L)** Post-op probe adjustment. A large 0.15 - 0.3 mm adjustment is made immediately after surgery.

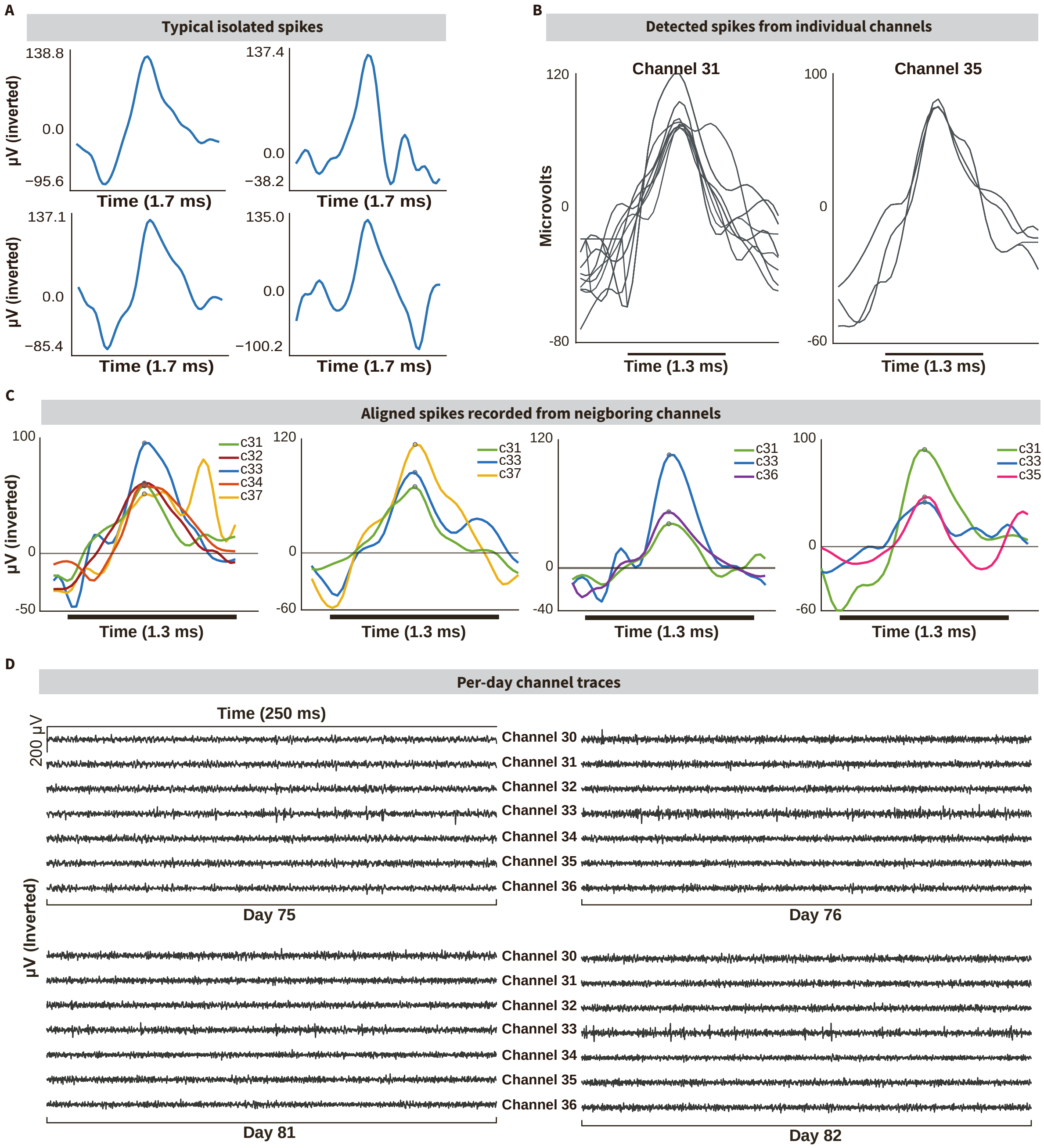

Figure S4. Additional electrophysiological data, related to **Figure 6. (A)** Isolated spike waveforms detected from individual channels. **(B)** Spikes which were detected across individual channels, overlaid and aligned to show the average waveform. **(C)** Spikes detected across various neighboring channels. Spikes were aligned to the highest-value sample (circled) and overlaid to show that the general waveform shape is conserved across at least three channels corresponding to neighboring electrodes. This process can be expanded to enable spike sorting. **(D)** Continuous traces from channels 30 – 36 recorded 75, 76, 81, and 82 days after surgery. -60 µm adjustments were made on days 74 and 81. Spike detection was performed with a –80 µV threshold, and only spikes with an amplitude equal or greater to 1 standard deviation above the mean were counted. Spikes remained detectable on each of these days, showing that recordings were stable and could be used for further analysis several days after surgery.

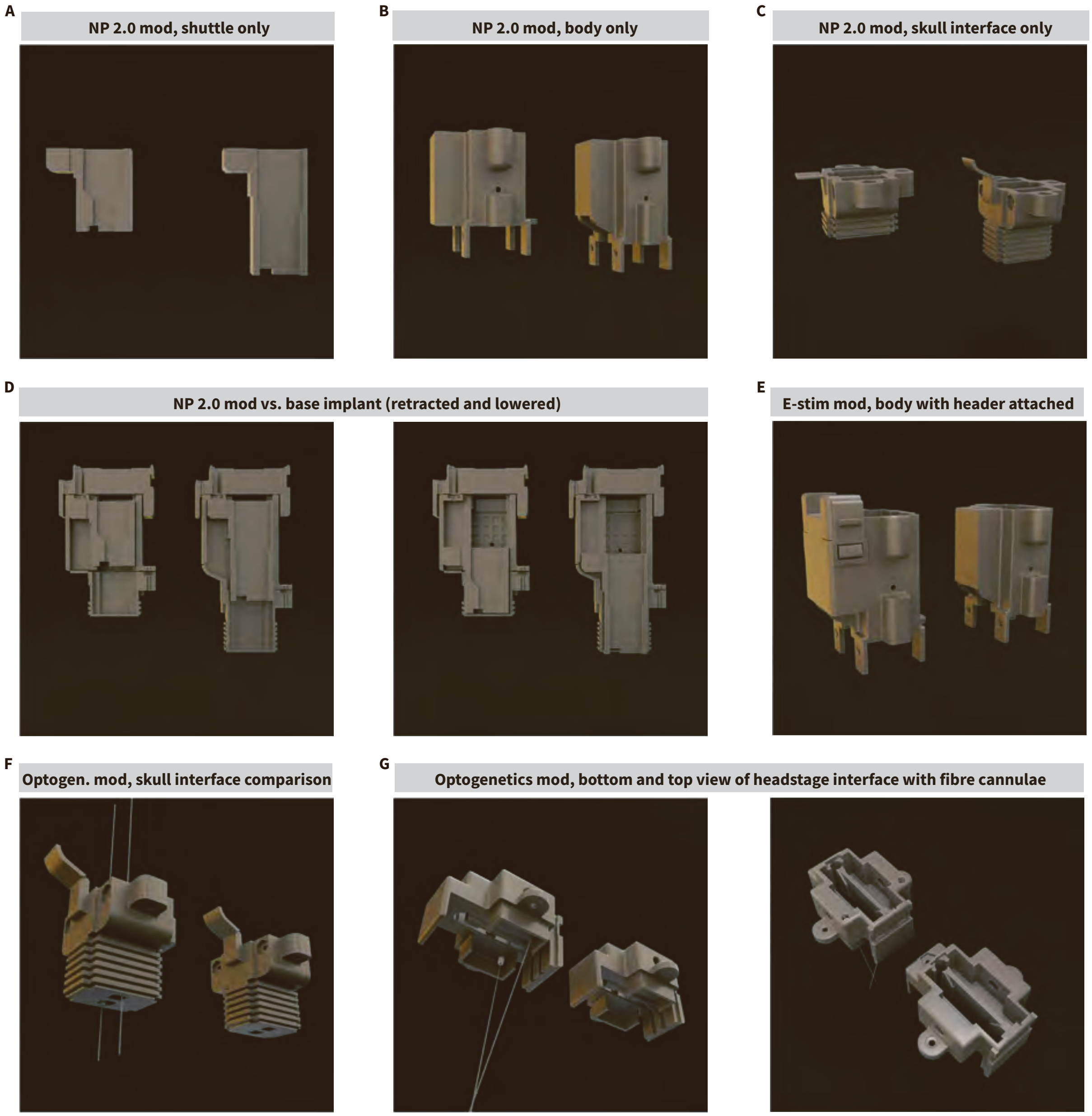

Figure S5. Extended proof-of-concept modifications, related to **Figure 7. (A - C)** Side-by-side comparisons of each of the modified pieces that make up the Neuropixels 2.0 (NP2.0) modified implant; modified pieces are on the left, and the original pieces are on the right. The shuttle (A) was shortened, and the shuttle bed was altered to accommodate the shorter NP2.0 probe. The implant body (B) was also shortened slightly, and the drive screw rail was elongated to ensure that the screw mechanism and shuttle are covered. The skull interface (C) was also shortened, and the drive screw rail cover was modified to fit the new shape seen on the implant body. **(D)** Side-by-side bisected view of the NP2.0 and original implants showcasing the shuttle's ability to retract into the implant (left panel) and be lowered fully (pictured right). **(E)** With the addition of pin headers to implant body for the electrical stimulation implant modification, we have also designed a header connector (left) with a latch that secures the connector in place and may resist some pulling. **(F)** Bottom view of the optogenetic mod skull interface. The cannulae are inserted from the inside of the skull interface piece and hold an optical fiber 1.25 mm away from the probe laterally, and 0.5 mm away from the probe tip vertically. **(G)** Bottom (pictured left) and top (pictured right) views of the headstage interface piece made for the optogenetic mod. Cannuale are inserted into the piece to direct the optical fiber through one side of the implant body to avoid excessive bending and possible breakage. On the top part of the piece, the recessed cannulae create a connector receptacle for optogenetic equipment.

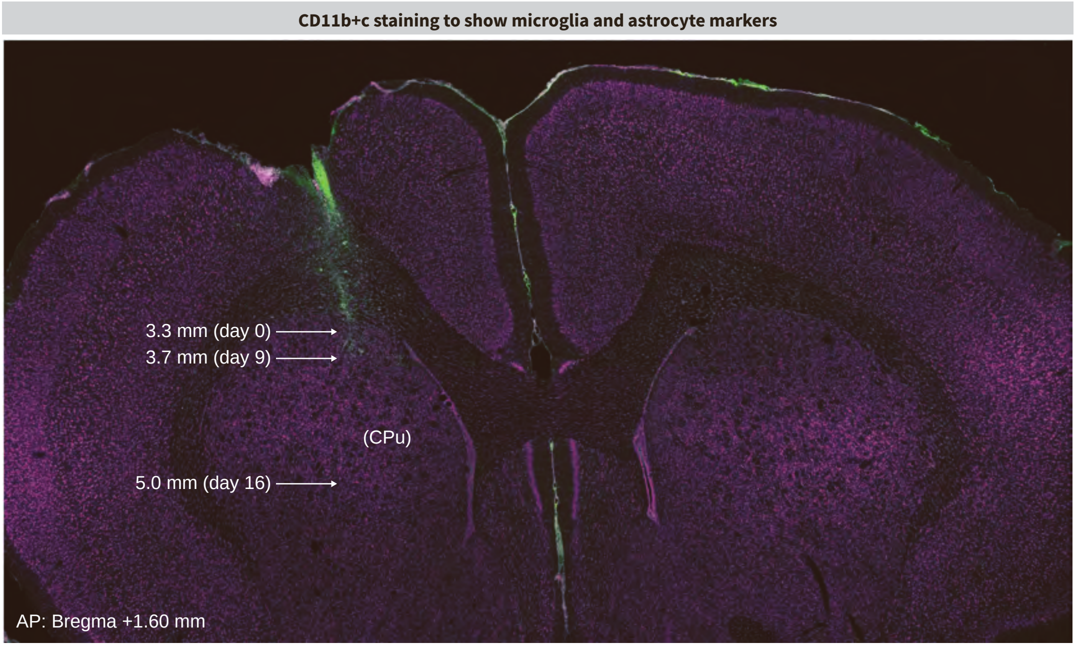

Figure S6. CD11b+c immunohistochemistry, related to **STAR Methods.** Foreign body immune response was assessed through CD11b+c staining to identify microglia and astrocyte markers around the probe shank. A glial scar can be observed along the track left by the probe shank from the initial implantation depth (day 0) through the first week post-implantation ending at day 9. Adjustments made in the second week left minimal markers.

Methods S1. Surgery and home cage modifications protocol, related to STAR Methods.

Section 1: Surgical procedures

Step 1: Surgical Prep

1. Construct custom rat cage (**see 'Section 3: Custom extra-tall cage'**)
2. Sterilize tools before surgery
3. Sterilize implant with alcohol, then dip in saline
4. Follow PPE guidelines
5. Check and fill isoflurane and oxygen tank
6. Weight charcoal tanks, document weight:
7. Turn on heating element: heating pad
8. Weigh the animal

Step 2: Anaesthesia / Shaving

1. Administer Ketamine Cocktail (ketamine (100 mg/kg) – xylazine (10 mg/kg) solution) through an Intraperitoneal (IP) injection. Dosage: *0.1 ml/ 100 gm* or *0.05 ml/100 gm*
    a) Conduct a toe pinch to ensure that the animal is fully anesthetized
2. Write down the total amount administered including boosts. Boosts should be administered intramuscular and should be ≤ 0.01 ml.
3. Anaesthesia: induction: 2%, maintenance: 1-1.5%, oxygen flow: 2-2.5 L/min
4. Put PURALUBE ointment on eyes to lubricate and protect them. Do this continuously throughout the surgery.

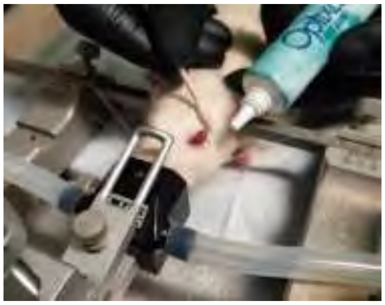

5. Shave scalp, from ears to near the nose *or* use Nair hair remover cream to remove hair from the desired area

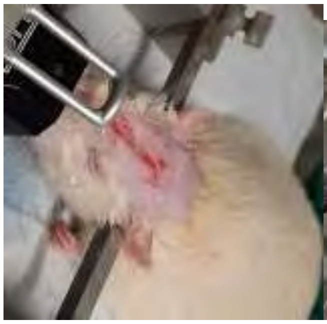 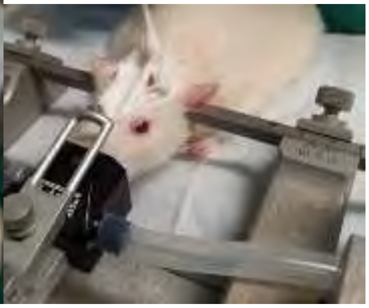

6. Secure animal in stereotaxic frame.
) Make sure the animal can say “yes” but not “no” ensuring that the skull does not move.
7. Insert the ear bars (<1mm difference) L=__________ R=__________
8. Clean surface with 70% alcohol, then iodine. 3X repeat
9. PURALUBE ointment on eyes AGAIN to prevent corneal drying
10. Use a thermometer to check the temperature of the rat before opening. Continue to check.

Step 3: Begin surgery

1. Inject 1ml of Bupivacaine subcutaneously around the incision site and massage it in

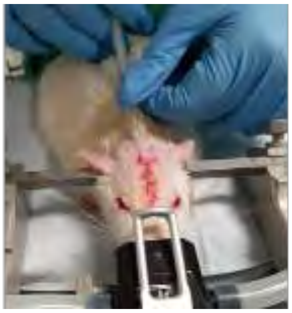

2. Expose cranium by making a midline anterior-posterior surgical incision, from the nasal bone to the end of skull with scalpel blade
3. Carefully push back and remove any connective tissue to fully expose the skull.

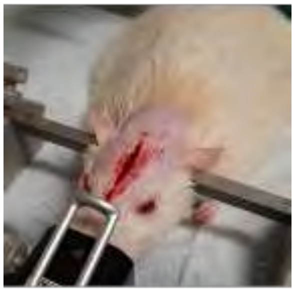 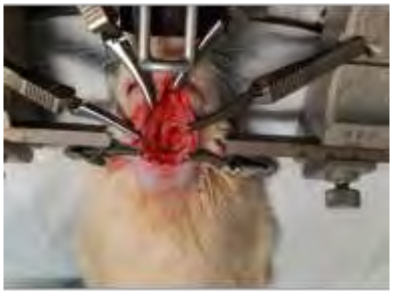

4. Remove any remaining connective tissue and with surgical bulldog clamps hold back the tissue

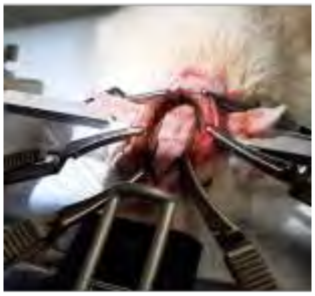

5. Use h2o2, styptic powder, or cold saline to clean and stop bleeding

Step 4: Screws

1. **FLAT SKULL**: Locate Bregma and Lambda and ensure < 0.5 mm difference between the two

Bregma	Lambda
DV:	DV:
ML:	ML:
AP:	AP:

2. Locate and mark the implant hole based on the coordinates below. Make a small indentation mark with a small ball-head 0.6mm drill bit once you locate where you will be implanting your probe.

AP (-1.2mm from Bregma):	ML (+2.5mm from Bregma):

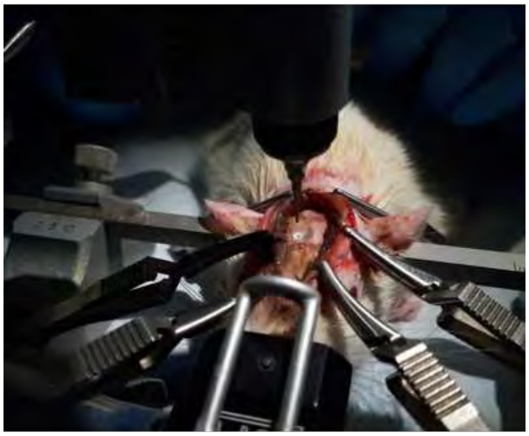

3. With a 1.5mm trephine micro drill bit go back to your target coordinates and drill a mark over your previous mark from Step 2.

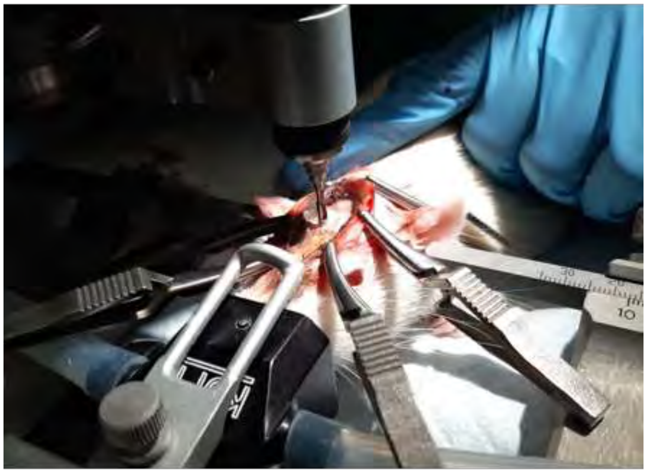

4. Temporarily lower the probe housing and place above the marked divot to ensure the screws are outside the area the housing will take up. You can mark the border around the probe housing with a surgical marker.

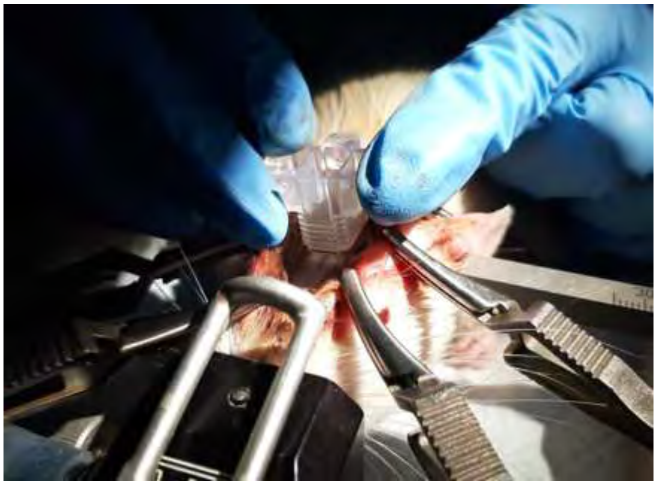

5. Plan where you will place your screws. NOTE: you will need 6-10 total screws. 1 screw will serve as the ground screw. This screw cannot be touching any other screws nor be near muscle or tissue as to reduce or avoid electromyographic interference during recordings.

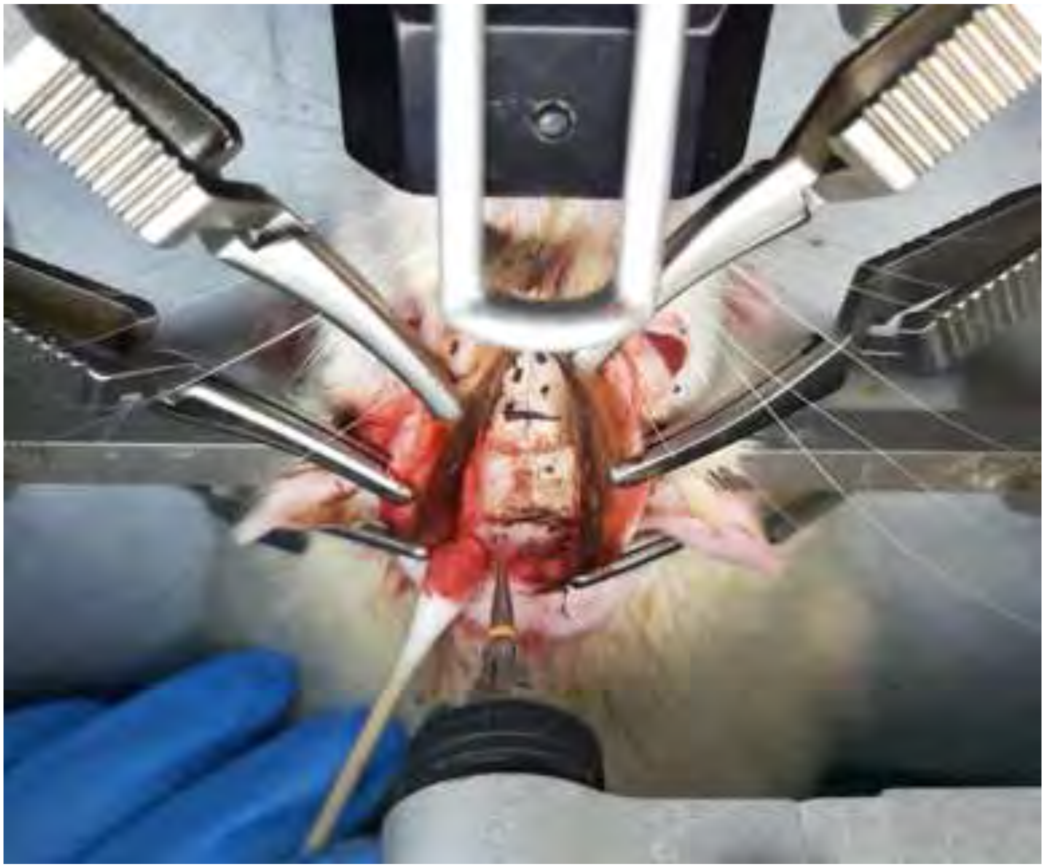

6. After you mark where you will be placing the screws, using a 0.6mm drill bit, make small holes that are deep enough for the screws to hold onto the bone but not deep enough to fully penetrate bone or dura mater. Once you have your hole, insert the screws using a small screwdriver and some pressure. Make sure to not screw in fully (as long as they are tightened and secured).

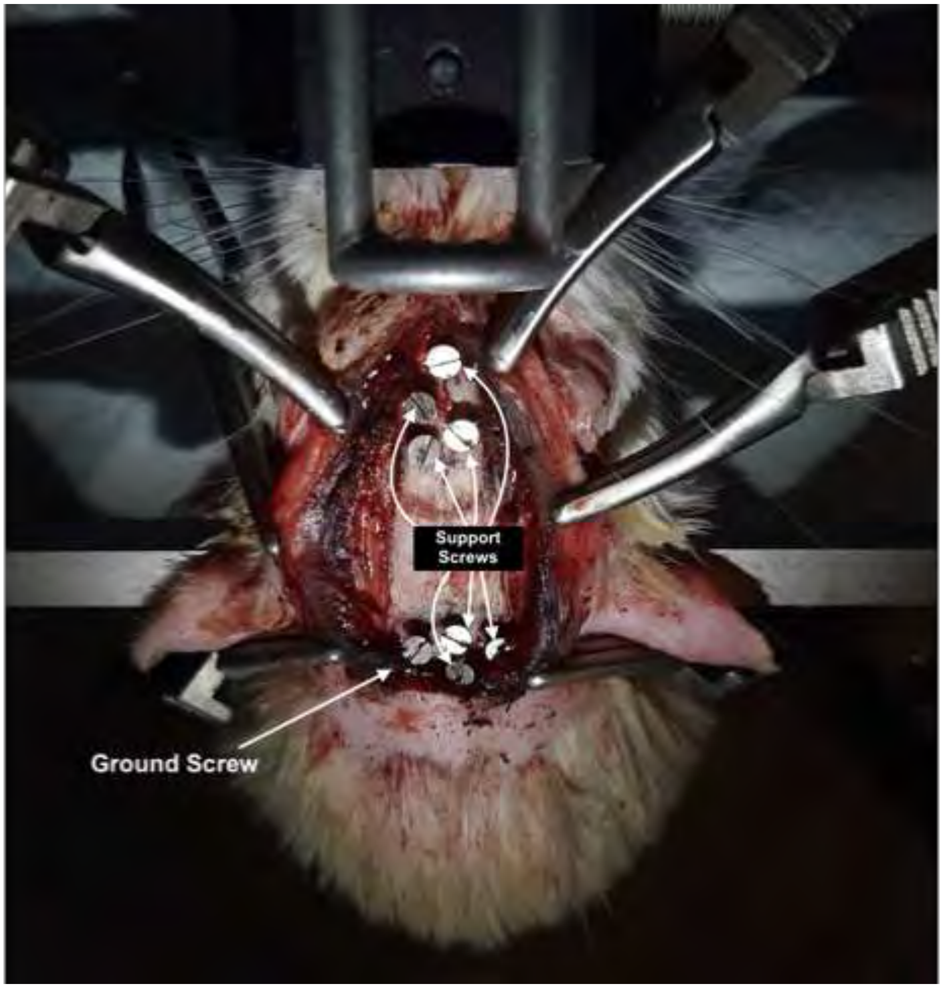

Step 5: Probe implant

1. Based on the Bregma, locate your coordinates

ML bregma ______ +2.5 =_______// AP ______ -1.2 = ________

   a) Locate Bregma, then move to coordinates.
   b) Mark the skull, then lift drill bit to ensure the smaller hole is in the middle of the marked craniotomy hole
2. With the 1.5mm trephine drill bit, carefully drill until the bone is translucent and barely attached on one side.
3. With small surgical tweezers carefully remove the bone. Save the piece to measure later.
4. Carefully remove any remaining dura mater using precision tweezers under microscope

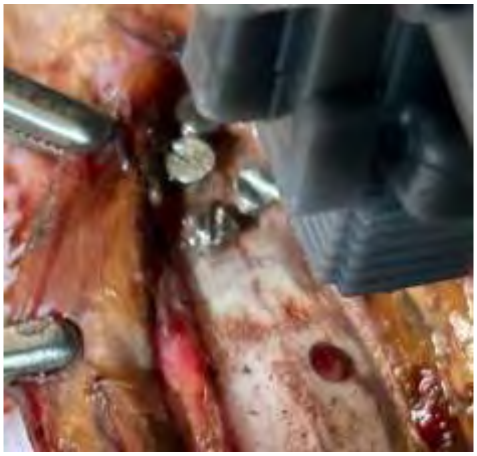

5. Inside of the craniotomy drop around 1-2 drops of 4 μg/mL dexamethasone is dropped into the top of the trephined hole, allowing for temporary softening of the brain, and reducing risks of inflammation

Step 6: Probe

1. Secure implant on the stereotactic arm and on the specialized implant holder
2. Locate your ground screw and screw the copper ground pad onto the skull. Careful to not break the wire connecting the ground pad to the implant

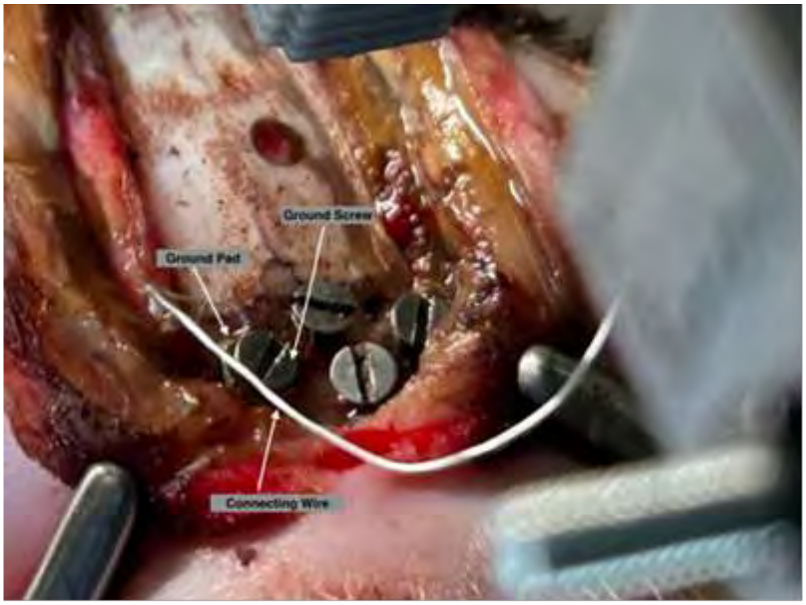

3. With the screwdriver make ~17 turns to bring out the probe approximately 5 mm
4. Locate bregma, then move to target coordinates

AP (-1.2mm from Bregma):	ML (+2.5mm from Bregma):

5. Measure the skull thickness by measuring the bone extracted from the trephine hole in **Step 5** with callipers. Take note of it.
    a) Although risky, this distance can also be calculated by subtracting the DV coordinate where the skull is located from the DV coordinate from where the surface of the brain is located. To do this, first expose the probe shanks to some arbitrary amount (f.e. 8 mm).
    b) Touch the probe shanks to a portion of the skull near the craniotomy while monitoring them with a surgical microscope. Take note of the DV coordinate on the stereotaxic device.
    c) Place the implant over the hole and zero at the Dura mater coordinate. Lower the implant until the shanks touch the brain. Take note of this DV.
    d) Subtract the DV from where the shanks touched the skull from the DV where the shanks touched the brain inside the craniotomy. This will be the skull thickness.
    e) Fully retract the probes again.
6. Place the implant over the hole and zero at the Dura mater coordinate
7. To ensure that the tip of the probe is implanted at a KNOWN depth, first expose the probe shank X mm by turning the drive screw counter-clockwise Y times (see formulae below or consult the surgical log excel sheet calculator).
    a. $X = (Skull\ thickness + padding) + DVinit$,
    Where DV_{init} is the desired initial implantation depth or *DV* (can be anywhere between 1.4 and 2.0 mm, depending on the brain region which is being targeted), and *padding* is the thickness of the bottom wall of the implant's skull interface. Typically, *padding = 0.5 mm*.
    b. $Y = X/\ (Drive\ screw\ pitch\ in\ mm)$.
    Drive screw pitch will likely be 0.3 mm if the materials in **Supplemental Note 4** are used.

DV(-3.5 from Dura)

WARNING: The probe shanks will be exposed to damage at this point. If you need to manoeuvre under the implant, do so with extreme care!

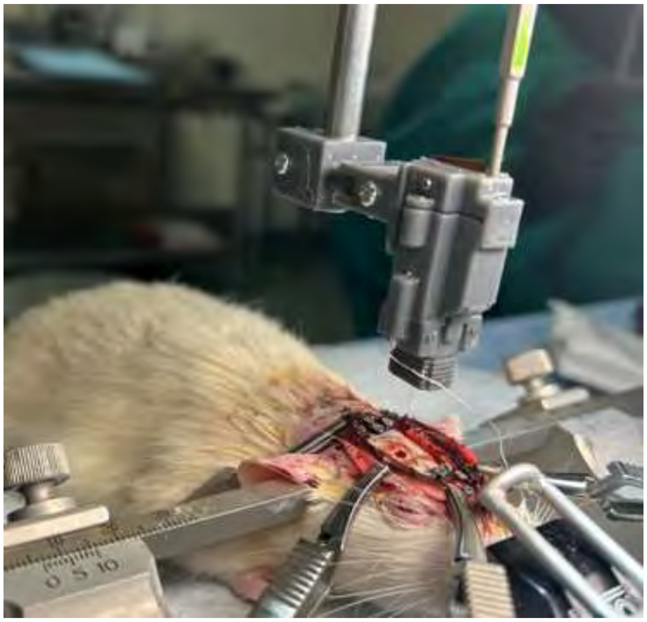

8. Add more dexamethasone and with a point swab or rolled up sterile paper towel bit, remove any excess liquid or blood
9. Make sure there is no dried blood or remaining dura and add more dexamethasone.
10. Using a microscope, make sure you can see both the tip of the probe and the surface of the brain.
11. Slowly lower the probe into the brain, but before you fully implant, stop at the top of the brain tissue and “dance” or “bounce” on it with the probe
    a) You are making sure that when you are dancing the probe is not bending. IF it does bend add more dexamethasone to irrigate the brain tissue.
    b) Once you can see the fragile probe is not bending, slowly lower it until the implant housing lays flat on the skull.
    c) Go back up, check the probe is still intact. If it is, repeat the “dancing” and irrigation steps and fully lower.

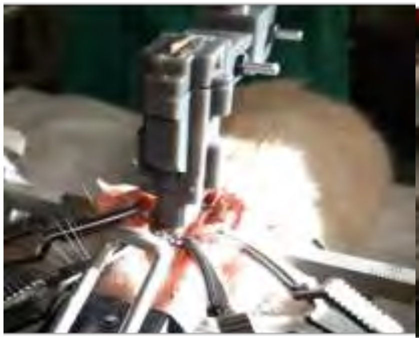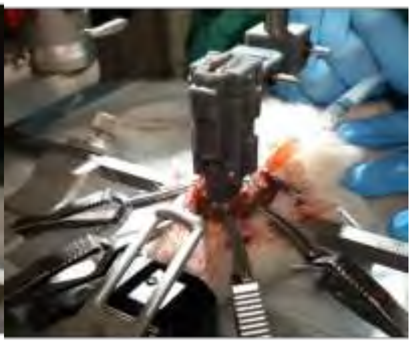

12. Once the implant housing is flat on the skull again, using a syringe with a 25” needle carefully put liquified sterile Vaseline into the probe area on top of the skull and within

the trephine hole to prevent the buildup of blood or other bodily fluids or tissues onto the probe

13. Wipe the excess around the exposed skull area with a cotton swab
14. Use 1-3 sticks of silver nitrate (activate it using saline) to put around the muscle tissue and the skull to limit re-growth.

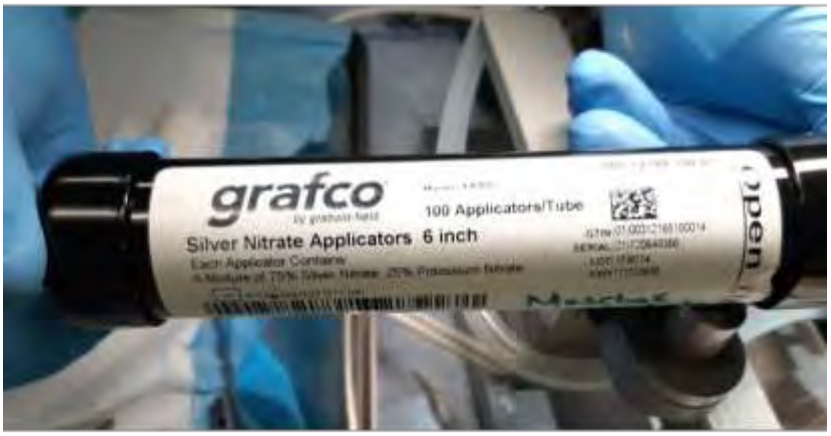

Step 7: Cement Metabond

1. Mix liquid-Metabond (5 drops blue liquid + 2 drops of Gold catalyst) with solid-Metabond (2 scoops of metabond powder)
2. Make sure the skull is clean and mostly dry and proceed to fully cover the skull with a thin layer of liquid-metabond. Let it dry.

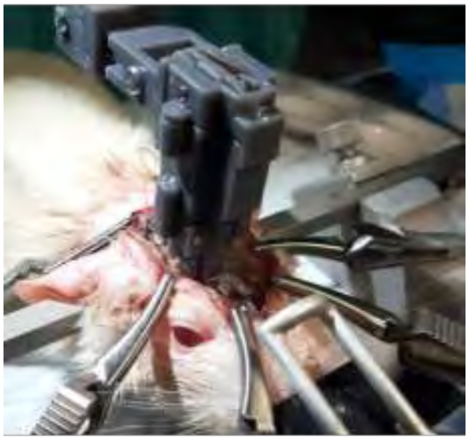

AVOID GETTING NEAR THE HOLES OR UNDER THE IMPLANT

3. Use the metabond mixture to build a layer on top of the skull
4. Let the cement fully dry (While waiting, cut their nails)

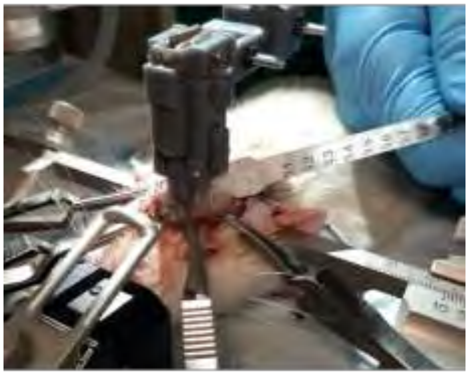

5. Dental Cement: Add a layer of cement above the metabond and layer by layer build a headcap around the implant, making sure that your layers dry before adding more cement.
6. Make sure the entire region is covered in cement (screws, any exposed skull, and the bottom ⅓- ⅔ of the implant housing). Be sure no hair is caught in the cement, and that no cement falls into the probe area.
7. When the cement hardens, loosen the screw and nut that hold the implant onto the holder and slide it off the implant by raising the stereotactic arm.

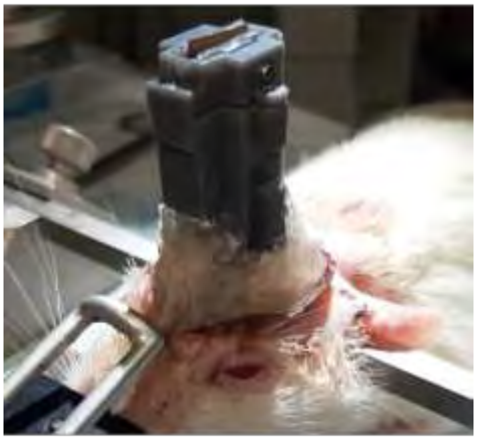

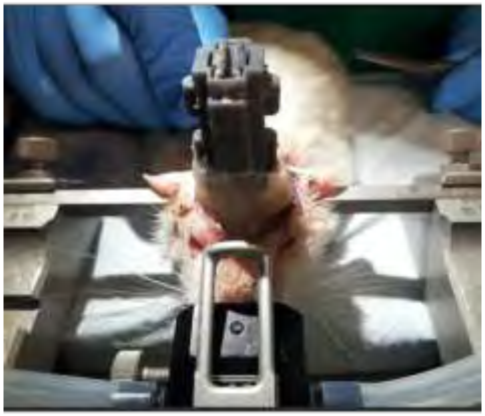

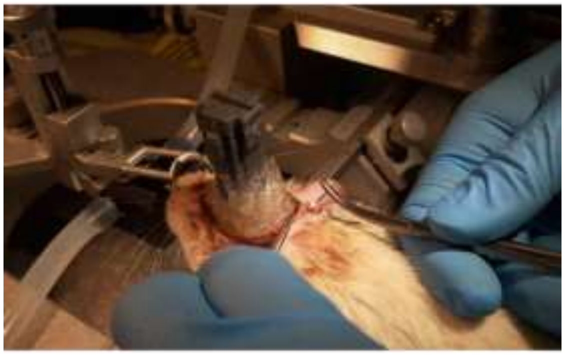

8. Once your cement layers have fully dried, carefully remove the implant holder
9. If there is excess skin do 2-3 sutures in the back <u>over</u> the cement headcap that you build using 5-0 silk sutures.
10. Check temperature = ____________ F

Step 8: Closing up

1. Lower the anaesthesia levels Oxygen= _________, Iso =_________
2. Rinse area with antibiotic lavage and chlorhexidine solution

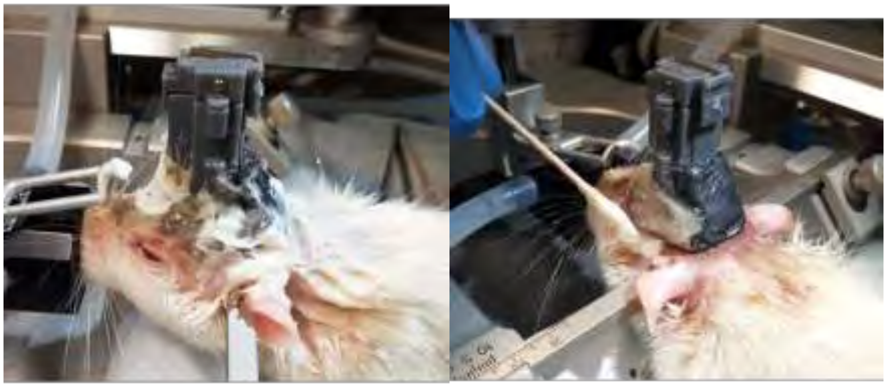

3. Add a mixture of triple antibiotic (neomycin, polymyxin B, and bacitracin) and chlorohexidine creams around the area and under the nails.

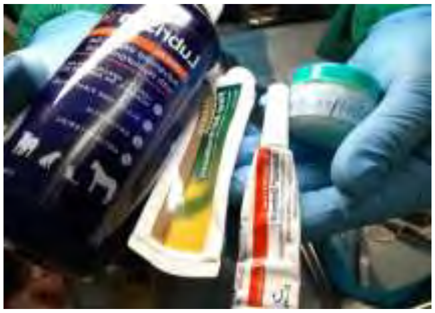

Step 9: Immediate post-op care and probe adjustment

- Inject Antibiotic and Meloxicam in 10ml of Ringer solution.
    - Inject the solution warmed (5ml on each side, **see Table S3.1 for dosing**)
    - Inject Rx for 3 additional days post-op and one extra day of antibiotic only in 10ml of Ringer (**see 'Section 4: Post-op care guidelines'** for additional first-week post-op care).

Enrofloxacin (Antibiotic)	**Antibiotic** Dose 5mg / 1 kg = x mg / kg	**Antibiotic** Concentration 22.7 mg / 1 ml = mg / x ml	**Antibiotic** Final volume:
Meloxicam (NSAID)	**Meloxicam** Dose 1mg / 1 kg = x mg / kg	**Meloxicam** Concentration 5 mg / 1 ml = mg / x ml	**Meloxicam** Final volume:

Table S3.1 | Antibiotic and NSAID injection dosing. Enrofloxacin and Meloxicam dosing chart to mix in with 10 ml Ringer.

- Using the *Kepler screwdriver*, lower the probe 100 - 200 µm (see surgical log excel sheet calculator).

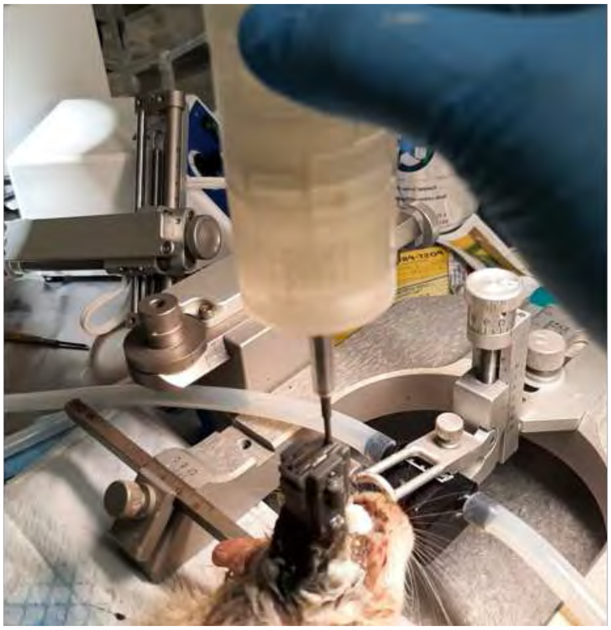

- Apply the mixture of creams (triple antibiotic: neomycin, polymyxin B, and bacitracin; and chlorohexidine creams) to promote healing and lower the risk of infection.
- Remove from anaesthesia. Time: __________
- Clean any blood from animal using saline
- Place in a new cage with surgical bedding, half on top of a heating pad
- Wait for the righting reflex, annotate the time the animal wakes up.
- Fill out post-op yellow card

Section 2: Potential complications and mitigation strategies for Neuropixels implant surgery

Damage to the Neuropixels probe during surgery can halt the procedure and compromise data integrity. To mitigate this, it is crucial to have a second implant prepared and ready for immediate use to avoid delays in cases where the probe shank fully breaks off. Probe damage can be minimized by ensuring it is fully retracted during steps where the probe is not in use, such as when screwing in the ground pad and ground screw. Retracting the probe into the implant during these steps protects it from accidental stress or impact. Proper handling and careful attention to each step of the surgery significantly reduces the risk of probe breakage.

If the connecting wire breaks while securing the ground pad, ensure soldering equipment, including a soldering iron, solder, and flux are available to repair the connection. Should this occur, the implant must be removed; this step must always be performed with the probe fully retracted to avoid further damage and before inserting the probe into the craniotomy hole. To further prevent the wire from breaking, be sure the wire is appropriately sized—around 50 mm in length—to allow manoeuvrability while minimizing the risk of tangling or breakage. The wire should be soldered parallel to the bottom of the ground pin on the skull connector for best stability. Adjust the implant's positioning using the stereotactic device to maintain alignment and stability during this process.

To prevent the implant from detaching post-surgery, ensure all connective tissue is meticulously removed during the preparation phase. Use silver nitrate sticks during surgery to prevent tissue re-growth underneath and around the implant. Additionally, applying silver nitrate around the cement 1–2 days post-op can further secure the implant if deemed necessary. This comprehensive approach ensures a stable and long-lasting implant, reducing the likelihood of complications.

Neuropixels implant surgeries present specific challenges that require careful preparation and real-time monitoring to ensure animal welfare and procedural success. Another critical complication is respiratory distress, including the cessation of breathing during surgery. To mitigate this risk, it is essential to maintain accurate ketamine dosing and to regulate isoflurane (iso) anaesthesia carefully. Isoflurane levels should be kept steady and low, not exceeding 2%, with oxygen flow consistently set at 0.5 L/min higher than the iso level. Continuous monitoring of respiratory rate is imperative, with adjustments to iso and oxygen levels made accordingly. Should an animal exhibit signs of waking at 2% iso, a 0.1 ml intramuscular (IM) boost of ketamine can be administered, followed by a reduction in iso once sedation stabilizes.

In the event of apnoea, immediate action is required. The animal should be removed from the nose cone, and a cardiac massage should be initiated while providing oxygen support. If breathing does not resume, cardiopulmonary resuscitation (CPR) should be started, including chest compressions and manual resuscitation with an oxygen pump, ensuring visible lung expansion. If these measures are ineffective, administer atipamezole hydrochloride (5.0 mg/mL stock solution) intramuscularly (IM) in the leg, avoiding the vein in the medial region. The dose is

determined by body weight and can be estimated using a mental shortcut: extract the hundreds digit of the weight in grams, multiply by two, and shift the decimal two places left (e.g., a 200 g animal: 2 × 2 = 4 → 0.04 mL). Alternatively, a precise calculation follows the equation Dose (mL) = 0.000184 × weight in grams, yielding 0.0368 mL for a 200 g animal, rounded to 0.04 mL. Both methods produce comparable results, with minor adjustments made as needed. If that does not work, as a final measure, humane euthanasia should be performed to minimize suffering.

Potential surgery complications, summary (❕)

1. (❕) Rat stops breathing during surgery.
    a. Immediately remove the rat from the isoflurane and begin CPR with assisted hand pump. Place the nosecone of the CPR pump on the rat's mouth and nose ensuring a tight seal. Press the hand pump to administer air. Ensure the rat's lungs can be seen inflating and deflating. Sometimes the air will enter the stomach – this means the rat is angled oddly. Ensure the rat is flat on their ventral side.
2. (❕) Rat starts waking up during surgery.
    a. Immediately cease any cutting/drilling. Check that the isoflurane line is connected properly, with adequate flow and dose being administered. If the rat wakes up fully, disconnect them from the bite bar before they damage their teeth. If the rat is barely waking up, hold the rat down and turn up the isoflurane briefly to administer a higher dose. <u>Ideally, you should always keep an eye on both the rat's physiological signs of anaesthesia AND the isoflurane/oxygen levels to prevent this.</u>
3. (❕) Rat is bleeding during surgery.
    a. This is expected, but depending on the level and location of bleeding it could be cause for concern.
        i. Bleeding at the skin
            1. Apply slight pressure using gauze and cold saline
            2. If bleeding continues, use a small amount of Kwik Stop Styptic Powder
        ii. Bleeding at the skull
            1. Apply slight pressure using gauze and cold saline
            2. If bleeding continues, use a small amount of Kwik Stop Styptic Powder. Note styptic powder can only be used before any craniotomies have been made.
        iii. Bleeding at the trephine hole
            1. With a point swab or rolled up sterile paper towel bit, remove any excess liquid or blood
        iv. Bleeding at the edges of the implant
            1. Wet a point swab with cold saline and with light pressure clean around the edges
4. (❕) The implant bent.
    a. Inspect the shank visually using a microscope. If no damage is visible and time allows, connect the implant to the system and test for damage before resuming implantation.
    b. If the implant is broken, use the backup (<u>*you should have a backup always*</u>*).*

Section 3: Custom extra-tall cage

Due to the size of the implant and risk of impact with traditional vivarium cages, we custom built an extra-tall cage for Neuropixels rats using the lower halves of two of our traditional cages. The steps below describe how these cages were built:

1. Stack the two cage halves open-side-down and *temporarily* tape them together with duct tape to make handling easier.
2. Test-fit the resulting box onto the vivarium rack and mark where the vivarium rack mating ports will need to be with a marker. The cage half with the markings will be the top half of the cage.
3. Begin by drilling a pilot hole using any drill bit smaller than 7/8 inch (about 2.22 cm) to guide the cut. Then, use a Dremel™ 545 Diamond Coated Cut-Off Wheel (which has a 7/8-inch diameter) to expand the hole to its final size. Ensure all burrs and melted acrylic plastic are removed for a clean finish.
4. The final hole will match the widest part (7/8 inch) of the green Tecniplast Green Line GR900 SealSafe Plus airflow mating ports. Insert the mating ports into the openings at the marked positions (**Fig. MS1a, top, green plastic and rubber grommets**), ensuring proper alignment with the vivarium rack (**Fig. MS1a, bottom**).
5. To secure the mating ports, apply hot glue between the outer edge of the mating port (7/8 inch) and the inner edge of the newly cut hole, forming a bond in the space between them. Focus on keeping the glue on the outside of the cage to prevent the rat from accessing it while ensuring a strong seal. Once the glue has fully set, set the cage aside.
6. To give the animal access to water, opt for water bottles with a bent waterspout or bend the spouts yourself. The bend angle should be greater than 90º, but less than 180º to allow water to flow through (**Fig. MS1b**).
    a. You may also omit bending the spouts; however, a bend can help with positioning the water bottles on the side of the modified cage in a way they don't protrude out as much.
    b. Place an extra rubber stopper along the length of each waterspout, 1-3 cm from the tip, to prevent the rat from getting stuck under the protruding metal tip, as this can be fatal. Set the bottles aside once prepared.
7. Using a drill/Dremel tool, make holes in the front side of the lower cage half (opposite side to where the mating ports are) to allow the water bottles to be inserted from the front of the cage (**Fig. MS1c**). *Unless you have a reason for not doing so, water bottles need to be placed in the front of the cage for the cage to fit in the vivarium rack.*
    a. If the cage allows, you may insert the bottles' waterspouts through an existing opening instead (**Fig. MS1d, arrow**).
8. Remove the tape from Step 1 and stack the two cage halves as they were in the previous steps. Tape the back edge (under the mating ports) of the two cage halves together to form a hinge (**Fig. MS1e**).

⚠ **Ensure that the sticky side of the tape is not accessible from the inside of the cage as this may pose a health risk to the animal.**

9. Add tape to secure the water bottles from the outside of the cage (**as shown in Fig. MS1c**), ensuring the rubber stoppers from **step 4b** are present to stop the spout from sliding into the cage too much. Only 1-3 cm of the spout should be inside the cage, adjust the rubber stopper to achieve this.

   ⚠ **Excessive protrusion is a risk for headcap arrestment and death of the rat.** *Excessive protrusion of waterspout is shown in **Fig. MS1d**. Ensure the waterspout is receded enough to prevent the implant from becoming lodged between it and the cage wall – this is a potential failure point. We use rubber stoppers along the waterspout to prevent it from reaching deep into the cage.*

10. Tape the post-op and rat information card holders to the front of the cage.
11. □**Clean the cage** with soap and warm water to remove any acrylic dust from drilling.
12. Add fresh, sterile low-dust surgical bedding to the inside of the cage. Fill the front of the cage with veterinarian-approved rat chow pellets. Add a cup of diet gel (wet food) topped with sugar pellets for easy nutrition while the rat heals.

(Figure MS1 on next page)

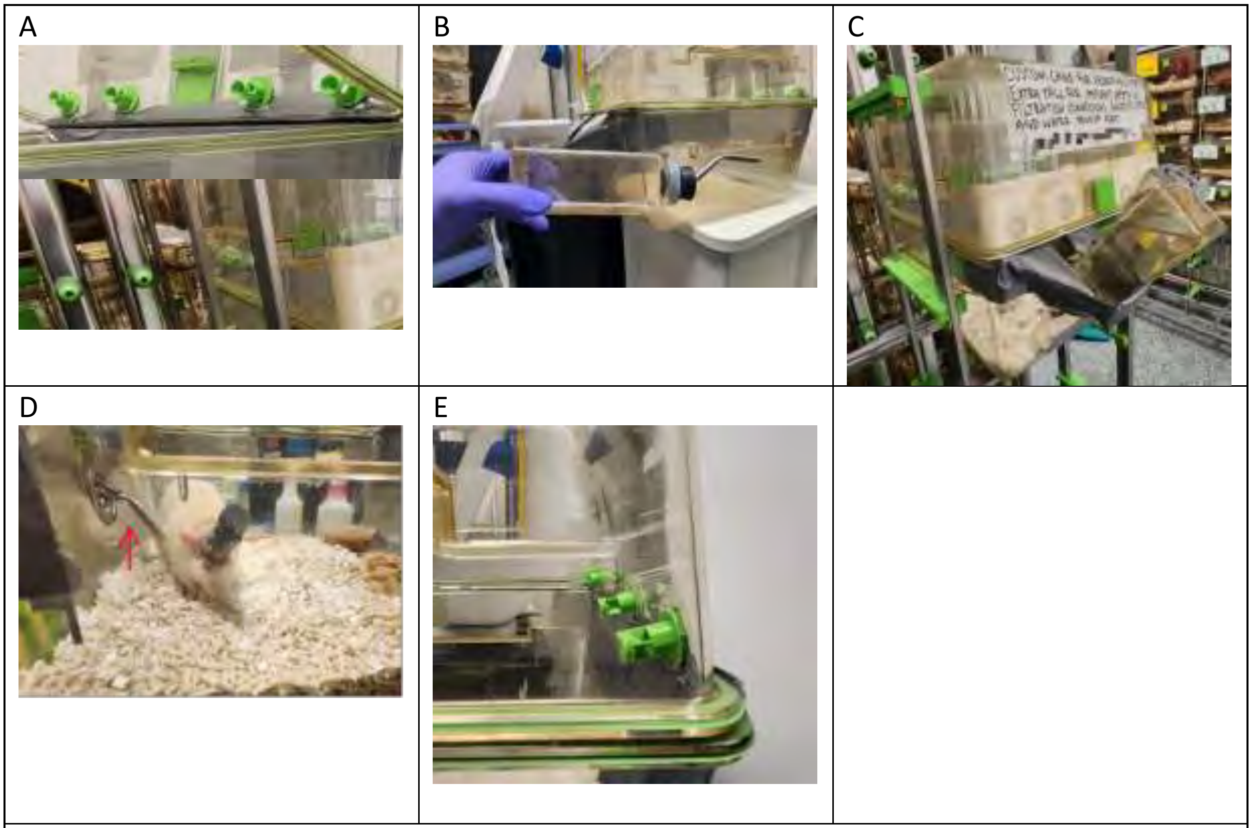

Figure MS1. Custom home cage, related to Methods S1. (A) Vivarium rack mating ports on custom cage. **(B)** Bending the water bottle spout to allow it to sit flusher with the outside cage wall. **(C)** Water bottles are positioned on the front of the cage to allow the custom cage to fit on the rack. **(D)** Water bottle spout introduced through a pre-existing entry hole on the cage. The figure shows too much of the spout inside the cage; ideally this length should be reduced to 1 – 3 cm by adding a rubber stopper along the length of the spout on the outside of the cage. **(E)** Duct tape hinge on the back of the cage. The sticky side of the tape is covered from the inside. Mating ports also pictured.

Section 4: Post-op care guidelines

4.1 First-week post-op care
As a general rule of thumb, cage bedding should be changed every 1 to 2 weeks depending on cleanliness. Due to the custom nature of the cage, it cannot be autoclaved without destruction, thus it must be manually deep cleaned with soap and warm water every few weeks.

⚠ **For the list items below, it is recommended to anaesthetize the rat with vaporized isoflurane prior to post-op care due to the risk of movement and to minimize pain.**

- Routinely inject gently warmed 10mL ringer with antibiotic Enrofloxacin and NSAID meloxicam (**Table S3.1**) every day for 3 days after the surgery date. Bolus is spread across two subcutaneous injections (5mL, 5mL) in the flank/back of the rat to accommodate for the 10mLs.
- Every day for a week, add a mixture of triple antibiotic (neomycin, polymyxin B, and bacitracin) and chlorohexidine creams around the implantation area and under the nails. (**Optional: add lidocaine cream for gentle numbing (at all stages of cream ideally – but not too often, lidocaine has its issues namely toxicity with excessive use.**).
    - (The rats nails should be trimmed during surgery to prevent excessive damage from grooming at the edges of the implant)
    - Lubrisyn (hyaluronic acid) may be added to the implantation area once the tissue has mostly scarred over to further promote healing.

4.2 Margin Care – 2-3 weeks after surgery
Margin care refers to caring for the margins of the implant's dental cement where the skin meets. Margin care is important to maintaining an infection-free site and prolonging the life of the implant and rat.

- Enrofloxacin (Enro) wash
- Benzocaine infusion
- Tweezer removal of gunk
- Enro wash 2
- Air Gun Drying
- Enro wash + silver nitrate stick (cauterization of open wounds; prevent bleeding, crusting, hair regrowth, skin regrowth)
- Saline wash to stop silver nitrate reaction
- Air Gun Drying 2
- Liquid Antibiotics + hyaluronic acid
- Air Gun Drying 3
- Optional: wait a few weeks and redo the procedure above before continuing, or:
    - Refill cement edges (only if necessary!)
